# Multifunctional Pan-ebolavirus Antibody Recognizes a Site of Broad Vulnerability on the Ebolavirus Glycoprotein

**DOI:** 10.1016/j.immuni.2018.06.018

**Published:** 2018-08-21

**Authors:** Pavlo Gilchuk, Natalia Kuzmina, Philipp A. Ilinykh, Kai Huang, Bronwyn M. Gunn, Aubrey Bryan, Edgar Davidson, Benjamin J. Doranz, Hannah L. Turner, Marnie L. Fusco, Matthew S. Bramble, Nicole A. Hoff, Elad Binshtein, Nurgun Kose, Andrew I. Flyak, Robin Flinko, Chiara Orlandi, Robert Carnahan, Erica H. Parrish, Alexander M. Sevy, Robin G. Bombardi, Prashant K. Singh, Patrick Mukadi, Jean Jacques Muyembe-Tamfum, Melanie D. Ohi, Erica Ollmann Saphire, George K. Lewis, Galit Alter, Andrew B. Ward, Anne W. Rimoin, Alexander Bukreyev, James E. Crowe

**Affiliations:** 1 Vanderbilt Vaccine Center, Vanderbilt University Medical Center, Nashville, TN 37232, USA; 2 Department of Pathology, Microbiology, and Immunology, Vanderbilt University Medical Center, Nashville, TN 37232, USA; 3 Chemical and Physical Biology Program, Vanderbilt University, Nashville, TN 37232, USA; 4 Department of Cell and Developmental Biology, Vanderbilt University, Nashville, TN 37232, USA; 5 Department of Pediatrics, Vanderbilt University Medical Center, Nashville, TN 37232, USA; 6 Department of Pathology, University of Texas Medical Branch, Galveston, TX 77555, USA; 7 Galveston National Laboratory, Galveston, TX 77550, USA; 8 Department of Microbiology & Immunology, University of Texas Medical Branch, Galveston, TX 77555, USA; 9 Ragon Institute of MGH, MIT, and Harvard, Cambridge, MA 02139, USA; 10 Integral Molecular, Inc., Philadelphia, PA 19104, USA; 11 Department of Integrative Structural and Computational Biology, The Scripps Research Institute, La Jolla, CA 92037, USA; 12 Department of Immunology and Microbiology, The Scripps Research Institute, La Jolla, CA 92037, USA; 13 The Skaggs Institute for Chemical Biology, The Scripps Research Institute, La Jolla, CA 92037, USA; 14 Department of Epidemiology, Jonathan and Karin Fielding School of Public Health, University of California, Los Angeles, Los Angeles, CA 90095, USA; 15 Department of Genetic Medicine Research, Children’s Research Institute, Children’s National Medical Center, Washington, DC 20010, USA; 16 Division of Vaccine Research, Institute of Human Virology, University of Maryland School of Medicine, Baltimore, MD 21201, USA; 17 Institut Nationale de Recherche Biomédicale, Kinshasa, Democratic Republic of the Congo

**Keywords:** ebolavirus, monoclonal antibodies, viral antibodies, neutralizing antibodies, cross protection, heterologous immunity, glycoproteins, Ebola hemorrhagic fever, epitopes, epitope mapping

## Abstract

Ebolaviruses cause severe disease in humans, and identification of monoclonal antibodies (mAbs) that are effective against multiple ebolaviruses are important for therapeutics development. Here we describe a distinct class of broadly neutralizing human mAbs with protective capacity against three ebolaviruses infectious for humans: Ebola (EBOV), Sudan (SUDV), and Bundibugyo (BDBV) viruses. We isolated mAbs from human survivors of ebolavirus disease and identified a potent mAb, EBOV-520, which bound to an epitope in the glycoprotein (GP) base region. EBOV-520 efficiently neutralized EBOV, BDBV, and SUDV and also showed protective capacity in relevant animal models of these infections. EBOV-520 mediated protection principally by direct virus neutralization and exhibited multifunctional properties. This study identified a potent naturally occurring mAb and defined key features of the human antibody response that may contribute to broad protection. This multifunctional mAb and related clones are promising candidates for development as broadly protective pan-ebolavirus therapeutic molecules.

## Highlights

• Broad human antibody recognizes a quaternary site of vulnerability on ebolavirus GP
• The antibody possesses pan-ebolavirus neutralizing and protective capacity
• The antibody mediates protection principally by direct virus neutralization
• The antibody uses several mechanisms for contributing to broad immunity

## Graphical Abstract

**Figure:**
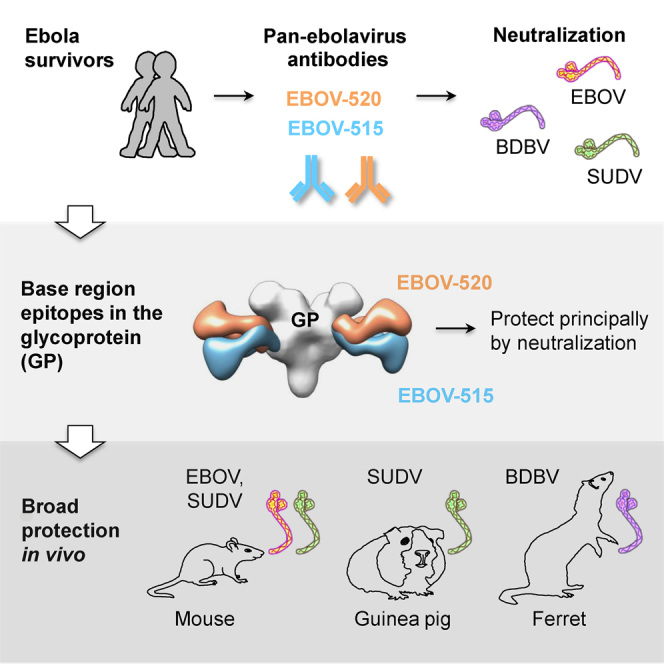

## Teaser

Gilchuk et al. isolated and characterized new broadly neutralizing human monoclonal antibodies active against all three clinically relevant ebolaviruses. Some potent antibodies bind to the glycoprotein base region, act principally by direct virus neutralization, and exploit several mechanisms for contributing to pan-ebolavirus protective immunity.

## Introduction

Ebola virus, a member of the *Filoviridae* family, causes severe disease in humans with 25% to 90% mortality rates and significant epidemic potential. There are no licensed ebolavirus vaccines or treatments. The largest 2013–2016 Ebola epidemic in West Africa, with a total of 28,646 cases of Ebola virus disease (EVD) and 11,323 deaths reported (Coltart et al., 2017), highlighted the need to accelerate EVD therapeutics development.

There are five known species—*Zaire ebolavirus*, *Bundibugyo ebolavirus*, *Sudan ebolavirus*, *Tai Forest ebolavirus*, and *Reston ebolavirus*—which are represented, respectively, by Zaire (EBOV), Bundibugyo (BDBV), Sudan (SUDV), Tai Forest (TAFV), and Reston (RESTV) viruses. EBOV, BDBV, and SUDV are clinically relevant viruses that are known to cause lethal disease in humans (WHO, 2017). Infections with RESTV are usually asymptomatic in humans, and only one case of non-lethal human infection has been reported for TAFV (CDC, 2017).

The Ebola virus envelope contains a single surface protein, glycoprotein (GP), which forms a trimer. The GP protomer consists of two subunits, GP1 and GP2. The GP1 subunit has a heavily glycosylated mucin-like domain and a glycan cap, which shields the host receptor binding site (RBS) that binds to domain C of its endosomal receptor, the protein Niemann-Pick C1 (NPC1-C). The GP2 subunit contains the internal fusion loop (IFL) and stalk and is anchored into the viral membrane by a transmembrane domain (Lee et al., 2008). The GP is solely responsible for viral attachment to the host cell, endosomal entry, and membrane fusion (Lee and Saphire, 2009), and thus it is also the major target for neutralizing monoclonal antibodies (mAbs) and vaccine design.

An experimental therapeutic mAb mixture, ZMapp, comprising three murine-human chimeric EBOV GP-specific mAbs (2G4 and 4G7 recognizing the base region and c13C6 recognizing the glycan cap), fully protected non-human primates (NHPs) from lethal EBOV challenge (Qiu et al., 2014). This cocktail also exhibited activity when used as treatment of EVD in humans in incomplete clinical trial testing during the recent epidemic (PREVAIL II Writing Group et al., 2016). ZMapp mAbs bind only to EBOV, however, and do not recognize BDBV or SUDV. We and others have isolated hundreds of new ebolavirus GP-specific mAbs from EBOV or BDBV survivors since the last EVD outbreak. New mAbs have been described that recognize diverse antigenic sites on GP, including epitopes on the glycan cap, the IFL, the GP1 head, the GP1/GP2 interface, the RBS, and the stalk (Bornholdt et al., 2016a, Bornholdt et al., 2016b, Corti et al., 2016, Flyak et al., 2016, Howell et al., 2016, Keck et al., 2015, Misasi et al., 2016, Pallesen et al., 2016, Wec et al., 2017, Zhao et al., 2017). Most of the mAbs isolated to date neutralize only one or two ebolavirus species. There is a medical need for mAb therapeutics that exhibit a pan-ebolavirus pattern of breadth, because the nature of future EVD outbreaks cannot be predicted. Recently, investigators identified the IFL as a site of vulnerability on GP, and they reported the isolation of three rare broadly neutralizing mAbs (bNAbs) that also possessed protective capacity against EBOV, BDBV, and SUDV. One antibody was derived from the B cells of immunized NHPs (Zhao et al., 2017), and two others were isolated from the B cells of a human survivor (Wec et al., 2017). These studies demonstrate that rare bNAbs against ebolaviruses are generated in response to natural infection or vaccination. Identifying potent bNAbs that can resist the emergence of viral escape mutants, and systematic analysis to define unique molecular and immunological features that mediate broad protection by these antibodies, are important next steps for rational selection of therapeutic mAb candidates.

Here, we isolated two human bNAbs, designated EBOV-515 and -520, that bound to sites of vulnerability on ebolavirus GP and mediated protection against EBOV, BDBV, and SUDV. In-depth analysis of the mechanism of action revealed key features that contributed to the broad reactivity, neutralization, and protection mediated by these mAbs. Together, the findings suggested high promise for these newly identified human mAbs as candidate pan-ebolavirus therapeutics.

## Results

### A Small Subset of mAbs Mediate Broadly Reactive Responses in Human Survivors of EVD

**Figure 1:**
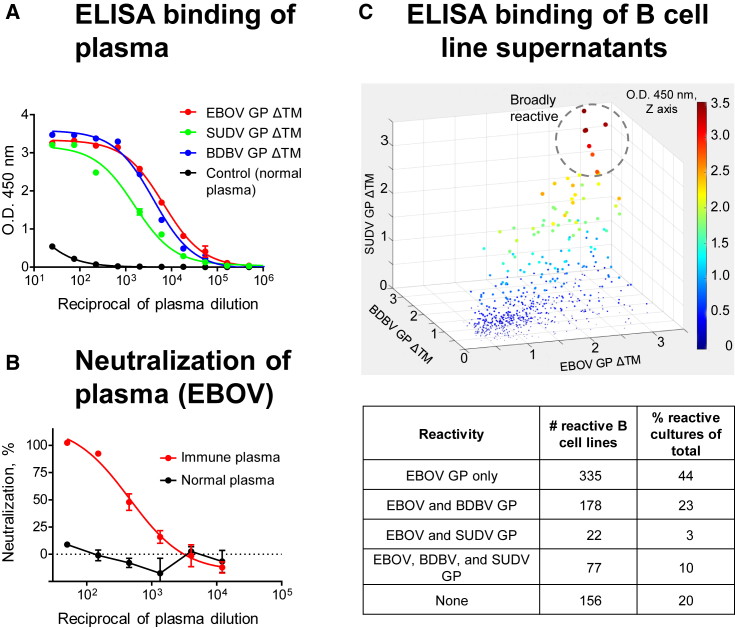
A Small Subset of Potent mAbs Isolated from B Cells of Survivors of EVD Recognize EBOV, BDBV, and SUDV GP (A) Binding of Abs in donor plasma to EBOV, BDBV, and SUDV GP ΔTM was assessed by ELISA. (B) Neutralization activity of donor plasma was determined using EBOV. (C) Binding of Abs in the supernatants of individual *in vitro* expanded B cell cultures (shown with dots) to EBOV, BDBV, or SUDV GP ΔTM was assessed by ELISA. Shown are data for a survivor of the DRC EVD outbreak. Mean ± SD of triplicates are shown, and data are representative of two independent experiments in (A) and (B). See also Table S1.

Plasma from 16 human survivors of the 2014 EVD outbreak in the Democratic Republic of the Congo (DRC) and one survivor of the West African 2013–2016 EVD epidemic were assessed for cross-reactivity against recombinant EBOV, BDBV, and SUDV GP lacking a transmembrane domain (GP ΔTM) to identify survivors that most likely have circulating memory B cells encoding bNAbs. Plasma from two survivors showed the highest activity to all three GPs by ELISA and also neutralized live EBOV (Figures 1A and 1B and unpublished data). Peripheral blood mononuclear cells (PBMCs) from these two donors were used to generate >600 EBOV GP-reactive lymphoblastoid B cell lines (LCLs). More than half of these EBOV-reactive LCLs produced Abs that bound to GP ΔTM of at least two ebolavirus species, and >10% bound in ELISA to EBOV, BDBV, and SUDV ΔTM GP (Figure 1C). LCLs producing broadly reactive Abs were used to generate B cell hybridomas. To identify hybridomas secreting bNAbs, their supernatants were assayed for neutralizing activity against live EBOV, BDBV, and SUDV. From two survivors, we isolated 16 broadly reactive mAbs with unique sequences (Table S1), three of which (designated EBOV-442, -515, and -520) neutralized all three viruses. These data suggested that EBOV infection elicited diverse B cell response including many clones secreting mAbs that bond to heterologous ebolavirus GPs, although only a small subset of those mAbs mediated cross-neutralizing responses.

### MAbs EBOV-515 and -520 Potently Neutralize EBOV, BDBV, and SUDV and Confer Protection against EBOV

**Figure 2:**
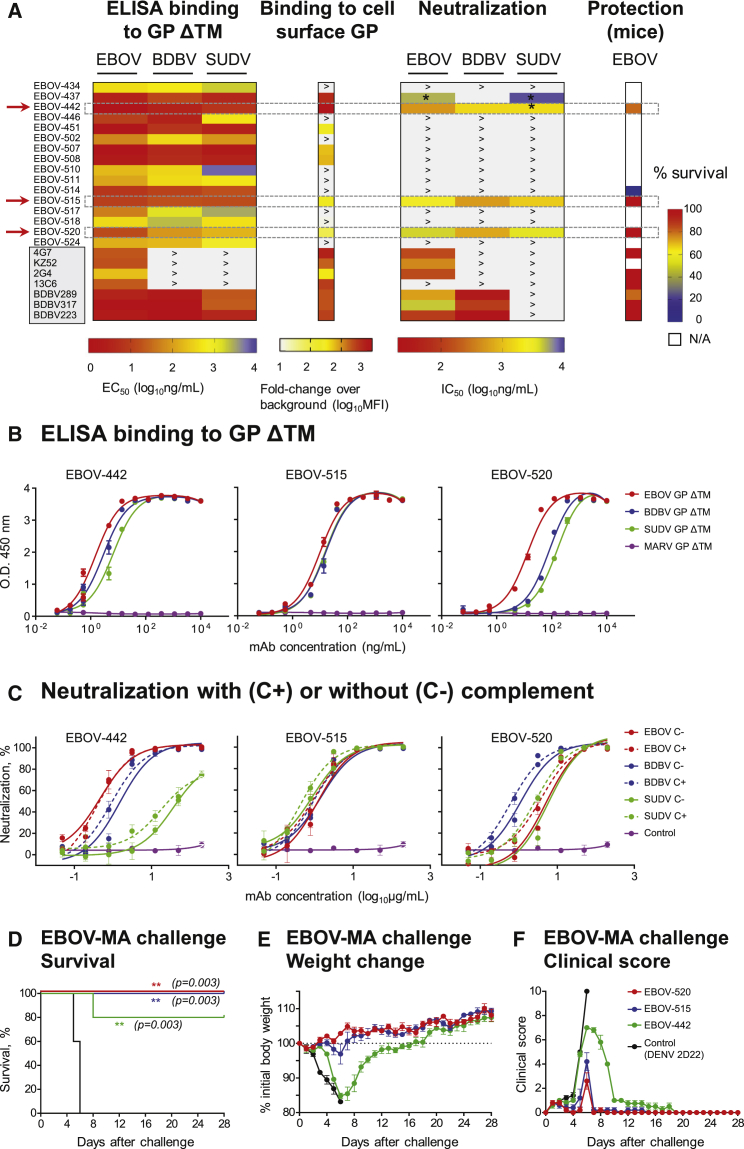
MAbs EBOV-515 and -520 Potently Neutralize EBOV, BDBV, and SUDV and Confer Protection against EBOV (A) Heatmap chart summarizing binding, neutralizing, and protective capacity of newly isolated or previously described (shaded box) mAbs. The red arrow indicates bNAbs. MFI, mean fluorescence intensity; ^∗^ indicates incomplete (<100%) virus neutralization at highest tested Ab concentration (200 μg/mL); > indicates activity was not detected at the highest mAb concentration tested (10 μg/mL for ELISA or 5 μg/mL for cell surface GP binding or 200 μg/mL for virus neutralization); N/A, not assessed. Protection data by known mAbs are from previous reports and included here for comparative purposes. (B) Binding of mAbs EBOV-442, -515, or -520 to EBOV, BDBV, or SUDV GP ΔTM was assessed by ELISA. (C) EBOV, BDBV, or SUDV neutralization by mAbs EBOV-442, -515, or -520. (D–F) *In vivo* efficacy of bNAbs against EBOV that assessed by survival (D), weight change (E), and clinical score (F). C57BL/6 mice were challenged with mouse-adapted EBOV-MA, treated with indicated mAb at 1 dpi, and monitored for 28 days. Mean ± SD of triplicates are shown, and data are representative of 2–3 independent experiments in (B) and (C). Mean ± SEM are shown, and data represent one experiment with five mice per group in (D) to (F). ^∗∗^p < 0.01 (two-sided log rank test). See also Figures S1 and S2 and Tables S2 and S3.

To assess the potency of EBOV-442, -515, and -520, we compared their activity to that of the other broadly reactive mAbs from the panel or to previously described GP-reactive mAbs recognizing the base, glycan cap, or HR2/MPER region (Figure 2A; Flyak et al., 2016, Flyak et al., 2018, Murin et al., 2014). Dose-response binding curves for the newly identified bNAbs showed high levels of binding to EBOV, BDBV, and SUDV GPs ΔTM in ELISA, with half-maximal effective concentration (EC_50_) values ranging from ∼10 to 200 ng/mL (Figures 2A, 2B, and S1; Table S2). EBOV-515 and -520 potently neutralized EBOV, BDBV, and SUDV with half-maximal inhibitory concentration (IC_50_) values ranging from ∼400 to 5,000 ng/mL. EBOV-442 neutralized EBOV, BDBV, and to a lesser extent SUDV. Complement was not required for neutralizing activity *in vitro* (Figures 2A and 2C; Table S3). We next used a recently developed flow cytometric assay to further characterize binding of individual mAbs to EBOV GP expressed on the surface of Jurkat cells (Jurkat-EBOV GP), which have been shown to express a form of trimeric GP likely very similar to the native form on virion particles or naturally infected cells (Davis and Ahmed, personal communication). Only a fraction of mAbs in the panel that bound to the GP ΔTM also bound to Jurkat-EBOV GP, but this group included all neutralizing mAbs (Figures 2A and S2). The results showed that bNAbs EBOV-442, -515, and -520 all efficiently recognized a form of trimeric GP that is anchored in a membrane on transduced cells likely very similar to the native form on virion particles or naturally infected cells.

To determine the protective capacity of the mAbs *in vivo*, we first tested EBOV-442, -515, and -520 in mice, against the mouse-adapted EBOV (EBOV-MA). An irrelevant mAb DENV 2D22 (IgG1 isotype) that is specific to dengue virus envelope (E) protein (Fibriansah et al., 2015) was used as a control. EBOV-515 and -520 each conferred complete protection from death, weight loss, and disease when delivered at a 5 mg/kg dose 1 day after inoculation (1 dpi) with EBOV-MA (Figures 2D–2F). EBOV-442 protected poorly in mice. Together, the results demonstrated the high neutralizing potency of EBOV-515 and -520 against all three clinically relevant ebolaviruses, and high efficacy of monotherapy with these mAbs that conferred full post-exposure protection against lethal challenge with EBOV.

### EBOV-520 Mediates Protection Principally through Virus Neutralization

In addition to neutralizing activity, mAbs may possess Fc-mediated functional activities that contribute to protection *in vivo*. To assess these additional functions in our broadly reactive mAbs, we used antibody-dependent cellular phagocytosis (ADCP), antibody-dependent neutrophil phagocytosis (ADNP), natural killer (NK) cell activation, and antibody-dependent complement deposition (ADCD) assays. These assays used immobilized EBOV GP ΔTM to determine the capacity of bound mAb to activate human effector cells *in vitro*. Functional profiling of 16 broadly reactive mAbs from the panel revealed a diverse activation pattern (Figure S3A; Table S4). Neutralizing mAbs EBOV-515 (IgG1), -520 (IgG4), and -442 (IgG1) triggered ADCP and NK activation *in vitro*, suggesting that their Fc also could be engaged in interaction with innate immune cells *in vivo*.

As many IgG4 antibodies possess anti-inflammatory activity (van der Neut Kolfschoten et al., 2007), we determined the functional capacity for Fc-engineered variants of the EBOV-520. We expressed the EBOV-520 variable region in recombinant form with the human IgG1 isotype (rIgG1) and also as a LALA Fc mutant (rIgG1-LALA) that binds only weakly to human Fcγ-receptors (FcγR) and has diminished function (Hessell et al., 2007). We compared the activity of the variant IgGs using dose response curves in ADCP, ADNP, NK activation, and ADCD assays. The rIgG1 showed higher activity when compared to the WT IgG4, and no activity was detected by these assays for rIgG1-LALA (Figure S3B).

**Figure 3:**
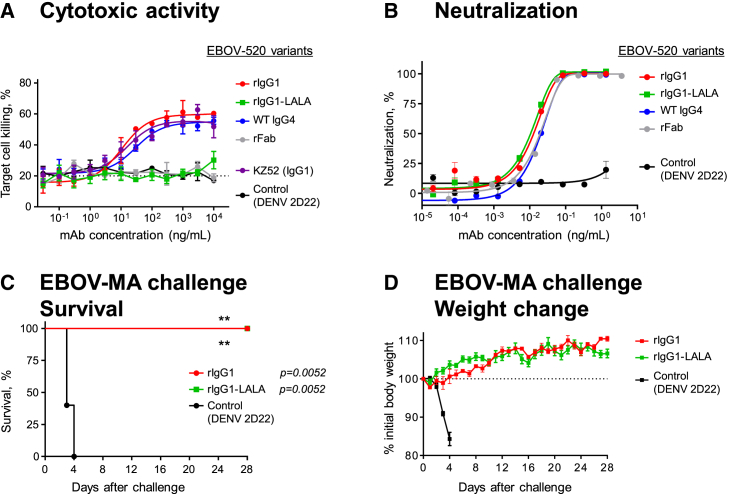
EBOV-520 Possesses Fc Region Effector Function Activity but Mediates Protection Principally through Virus Neutralization (A) *In vitro* killing capacity curves for engineered variants of mAb EBOV-520 that determined using SNAP-tagged EBOV GP-expressing 293F cell line as a target and human PBMCs as source of effector cells. Dotted line indicates assay background. (B) Neutralization of EBOV by engineered IgG heavy chain variants of mAb EBOV-520. (C and D) *In vivo* protective efficacy of EBOV-520 rIgG1 or rIgG1-LALA against EBOV. C57BL/6 mice were challenged with EBOV-MA, treated with indicated mAb in 1 dpi, and monitored for 28 days. Mean ± SD of triplicates are shown, and data are representative of two independent experiments in (A) and (B). Mean ± SEM are shown, and data represent one experiment with five mice per group in (C) and (D). ^∗∗^p < 0.01 (two-sided log rank test). See also Figure S3 and Table S4.

The Fc-mediated activity assays above used solid-phase display of GP ΔTM. We next determined whether EBOV-520 had a capacity to engage human effector cells in a system with properly oriented full-length antigen displayed on a cell surface. We used a stably transfected EBOV GP-expressing SNAP-tagged 293F cell line as a target, with heterologous human PBMCs as source of effector cells to assess dose-killing response. The SNAP-tag is a self-labeling protein tag that allows specific labeling of a target cell line with the SNAP-Surface Alexa Fluor-647 fluorescent dye, facilitating detection of effector cell-mediated killing activity by flow cytometry (Domi et al., 2018, Orlandi et al., 2016). The EBOV-520 rIgG1 and WT IgG4 showed dose-responsive cell killing, with activities comparable to that of the base region mAb KZ52 IgG1, while the low level of cell killing activity of rIgG1-LALA and rFab was similar to that of the control mAb of irrelevant antigen specificity (Figure 3A). These findings suggested that the bNAb EBOV-520 also may mediate function through the Fc region when expressed as rIgG1. All tested EBOV-520 variants, including the WT IgG4 and even monovalent rFab, showed a similar level of neutralizing activity to that of the full-length IgG (Figure 3B).

To evaluate whether Fc-mediated function was required for protection *in vivo* by EBOV-520, we tested rIgG1 and rIgG1-LALA variants in mice against EBOV-MA. The rIgG1 and rIgG1-LALA antibodies conferred complete protection when delivered at 5 mg/kg dose 1 dpi (Figures 3C and 3D), demonstrating that EBOV-520 mediated protection principally through virus neutralization. Together, these results suggested that direct virus neutralization alone could be sufficient to confer protection *in vivo* by a bNAb, although this type of antibody also may function through Fc-mediated activities that can be tuned by class switch of the isotype.

### EBOV-515 and -520 Use Several Mechanisms to Facilitate Virus Neutralization

We next sought to elucidate the molecular basis of neutralization by the three bNAbs identified above. Ebolavirus entry involves cathepsin-mediated cleavage of GP into cleaved GP intermediate (GP_CL_) in the endosome (Chandran et al., 2005). Cleavage removes the glycan cap and mucin-like domain of GP ectodomain, thereby exposing RBS for endosomal receptor NPC1 (Carette et al., 2011, Côté et al., 2011). Binding to NPC1 triggers structural rearrangements in GP2 that lead to membrane fusion (Spence et al., 2016).

**Figure 4:**
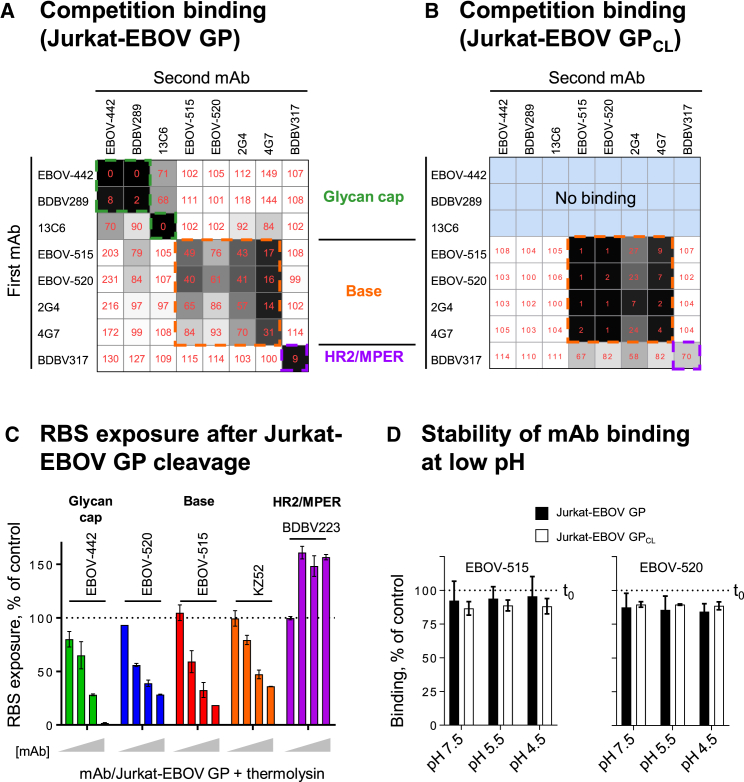
EBOV-515 and -520 Are Specific to the Base Region of GP and Possess a Capacity to Inhibit GP Cleavage (A and B) Identification of major antigenic sites for three bNAbs using a competition binding assay with intact Jurkat-EBOV GP (A) or thermolysin-cleaved Jurkat-EBOV GP_CL_ (B). Cells were incubated with the first unlabeled mAb and then with the second fluorescently labeled mAb. Binding of mAbs was analyzed by flow cytometry. Numbers indicate the percent binding of the second fluorescently labeled mAb in the presence of the first unlabeled mAb, compared to binding of the second mAb alone. (C) Capacity of bound mAbs to inhibit the exposure of the RBS after EBOV GP to EBOV GP_CL_. Varying concentrations of mAbs (1, 10, 20, or 40 μg/mL) were incubated with Jurkat-EBOV GP, followed by cleavage and measurement of the exposure of the RBS with fluorescently labeled RBS-specific mAb MR78 by flow cytometric analysis. Dotted line indicates % RBS exposure in the presence of control mAb DENV 2D22. (D) Stability of mAb binding to Jurkat-EBOV GP or Jurkat-EBOV GP_CL_ at neutral or acidic pH. Cells displaying GP or GP_CL_ on the surface were fixed, pre-incubated with fluorescently labeled mAb at neutral pH, and then exposed to neutral or low pH for 60 min. mAb binding was assessed by flow cytometry. Stability of binding was expressed as the percent of the control (maximal binding) when cells were analyzed immediately after staining and without exposure to the neutral or low pH. Mean ± SD of triplicates are shown, and data are representative of 2–3 independent experiments. See also Figure S4.

We first defined groups of neutralizing mAbs that bind to common major antigenic sites using a competition-binding assay with cell surface-expressed intact EBOV GP (Jurkat-EBOV GP) or the same cells that had been treated with thermolysin to mimic cathepsin cleavage to yield membrane-displayed GP_CL_ (Jurkat-EBOV GP_CL_). Cell surface-displayed GP_CL_ has utility for epitope mapping and mechanistic studies with mAbs, because it closely mimics the proteolytically primed receptor binding-competent GP intermediate that is generated in the host endosomal compartment during ebolavirus infection. EBOV-442 targeted the glycan cap (as shown by competition with glycan cap mAbs BDBV289 or 13C6) and it recognized only intact EBOV GP. The most potent bNAbs (EBOV-515 and -520) bound moderately to intact GP but strongly to GP_CL_, and they targeted the GP base (as shown by competition for binding with mAb 2G4 or 4G7) (Figures 4A and 4B). This finding suggested that EBOV-442 most likely acts prior to GP cleavage, while EBOV-515 and -520 may act either prior to or after cleavage.

We next assessed the capacity of the three bNAbs to inhibit GP cleavage. Jurkat-EBOV GP cells were pre-incubated with EBOV-442, -515, or -520. For comparison, we tested in parallel mAb KZ52 (base) with known inhibitory activity (Misasi et al., 2016) or a DENV 2D22 or the HR2/MPER-specific BDBV223 mAbs (negative controls). After cleavage, exposure of the RBS on GP_CL_ was measured by the level of binding of fluorescently labeled RBS-specific mAb MR78 that does not bind uncleaved EBOV GP (Flyak et al., 2015). EBOV-442, -515, and -520 inhibited cleavage in a dose-dependent manner, similarly to KZ52 (Figures 4C and S4A). EBOV-442 was the most efficient and completely inhibited GP cleavage at 40 μg/mL, while EBOV-515 or -520 revealed only partial inhibition at the same concentration. This finding supported our hypothesis further that the most potent base region-specific mAbs (EBOV-515 and -520) also may act after GP cleavage.

During infection, GP cleavage occurs in the acidified endosome with a pH estimated to be about 5.5. As antibody binding initially occurs at neutral pH during infection, we investigated the pH stability of the immune complex when mAb was pre-bound to cell surface-displayed EBOV GP or GP_CL_. Both EBOV-515 and -520 demonstrated stable association with GP or GP_CL_ at low pH, ranging from ∼84% to 96% of the total mAb bound at neutral pH and assessed before the exposure to low pH (Figure 4D). Stable binding of both bNAbs to GP or GP_CL_ in low pH compartments may allow them to act prior to or after the proteolytic priming step.

**Figure 5:**
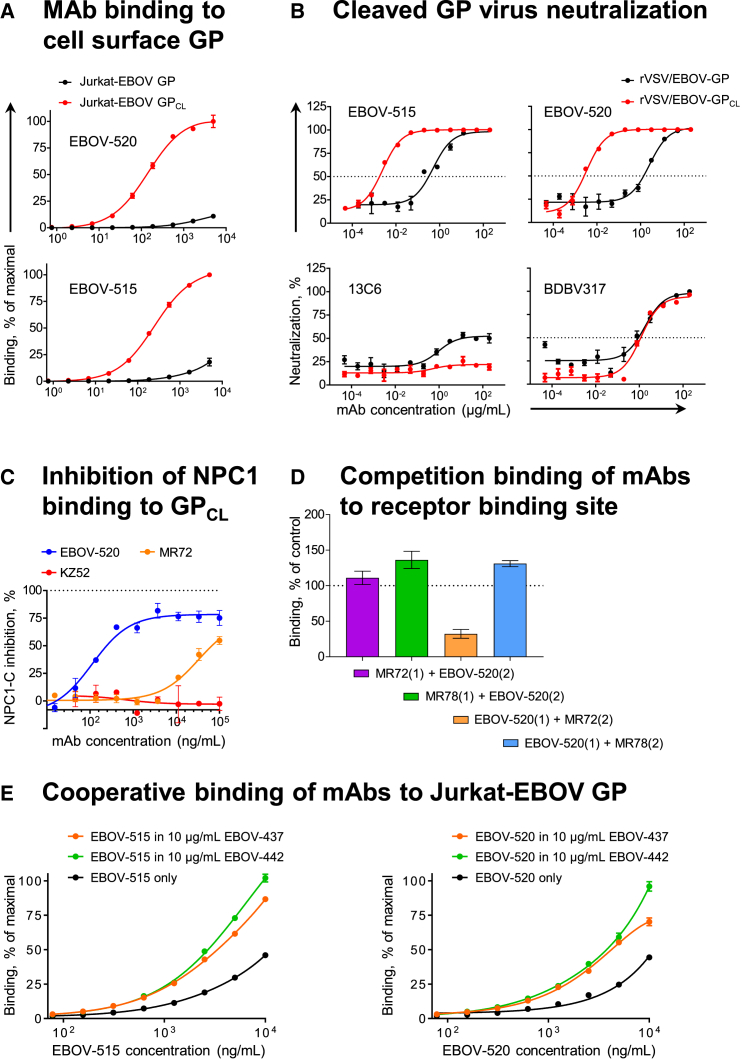
EBOV-515 and -520 Target Both Intact GP and Cleaved GP_CL_ Intermediate to Neutralize the Virus (A) Binding curves for EBOV-515 or -520 using Jurkat-EBOV GP or Jurkat-EBOV GP_CL_. Fluorescently labeled mAbs were incubated with cells, and binding was assessed by flow cytometric analysis. (B) Neutralization curves for EBOV-515 or -520 or control mAbs 13C6 or BDBV317 using rVSV/EBOV-GP or rVSV/EBOV-GP_CL_. (C) Capacity of mAbs to inhibit NPC1-C binding to GP_CL_. mAbs were incubated with Jurkat-EBOV GP_CL_, then with purified NPC1-C tagged with FLAG-epitope. Complexes were detected with anti-FLAG Abs by flow cytometry. (D) Competition binding of EBOV-520 with RBS-specific mAbs MR72 or MR78 was assessed using Jurkat-EBOV GP_CL_. mAb binding was analyzed by flow cytometry. Numbers indicate the percent binding of the second fluorescently labeled mAb (2) in the presence of the first unlabeled mAb (1), compared to binding of second labeled mAb alone (dotted line). (E) Binding curves of fluorescently labeled mAb EBOV-515 or -520 to Jurkat-EBOV GP in the presence of a fixed concentration of unlabeled mAb EBOV-437 or -442. Mean ± SD of triplicates are shown, and data in (A) and (C)–(E) are representative of two independent experiments. Data in (B) represent one experiment. See also Figure S4.

We next examined in more detail the interaction of base-specific bNAbs with cell surface-displayed GP_CL_. Binding of EBOV-515 or -520 to Jurkat-EBOV GP_CL_ was enhanced relative to binding of Jurkat-EBOV GP, while binding of BDBV317 (specific for an HR2/MPER epitope) to Jurkat-EBOV GP_CL_ was similar to that for Jurkat-EBOV GP (Figure S4B). Dose-response testing showed a dramatic increase (∼80- to 250-fold) in binding efficiency of EBOV-515 or -520 to GP_CL_ compared to intact GP (Figure 5A). This finding was concordant with the large (∼200- to 800-fold) increase in neutralizing potency for these mAbs against a replication-competent recombinant vesicular stomatitis virus (rVSV) displaying EBOV GP_CL_ (Figure 5B). We concluded that the antigenic site for these bNAbs is partially occluded on intact GP and more accessible after proteolytic priming to produce GP_CL_, which facilitates neutralization of cleaved virus in the endosome.

Given that these bNAbs were more potent against particles displaying GP_CL_, we next assessed their ability to inhibit binding of GP to its receptor NPC1. Binding of soluble NPC1-C to Jurkat-EBOV GP_CL_ was assessed in the presence of increasing concentrations of EBOV-515 or -520 (base), negative control KZ52 (base), BDBV317 (HR2/MPER), or positive control MR72 (RBS-specific, with NPC1-C-blocking activity) mAbs (Bornholdt et al., 2016a, Flyak et al., 2015). EBOV-520, similarly to MR72, exhibited dose-dependent inhibition of NPC1 binding to GP_CL_ (Figures 5C and S4C), suggesting that EBOV-520 may act by inhibiting receptor engagement.

The antibodies MR72 and MR78 target a hydrophobic pocket of the RBS that is exposed only on GP_CL_ of ebolaviruses or GP of the most divergent filovirus, Marburg (MARV) (Bornholdt et al., 2016a). Hence, we next tested whether EBOV-520 competes for binding with the RBS-specific MR72 or MR78. Pre-bound MR72 or MR78 did not block binding of EBOV-520 to GP_CL_ (Figure 5D), indicating that MR72 and EBOV-520 recognize non-overlapping epitopes. In contrast, EBOV-520 partially inhibited binding of MR72 (∼2.8-fold decrease) when GP_CL_ was pre-incubated with EBOV-520, suggesting that EBOV-520 could inhibit receptor binding indirectly by changing the conformation of the RBS.

We considered whether any of the isolated antibodies of differing epitope specificity could cooperate in binding to ebolavirus GP, since cooperativity has been reported previously (Howell et al., 2017). Thirteen non-competing mAbs from the panel were combined individually with EBOV-515 or -520 and then assessed for cooperative binding to Jurkat-EBOV GP (Figure S4D). Two neutralizing mAbs, EBOV-437 and -442 (from the glycan cap-specific group identified by competition binding), enhanced the binding of both EBOV-515 and -520 to intact GP ∼3- to 5-fold (Figure 5E). We concluded that such a cooperative binding effect could facilitate recognition of intact GP by bNAbs in polyclonal plasma or therapeutic antibody mixtures.

Together, our findings suggested several mechanisms that can contribute to broad neutralizing activity by an individual mAb (a property designated here as multi-functionality), which included inhibition of GP cleavage, inhibition of primed virus that displayed GP_CL_, inhibition of NPC1 receptor binding by an allosteric alteration, and cooperative binding to a vulnerable antigenic site on GP.

### EBOV-515 and -520 Recognize Distinct Vulnerable Epitopes in the Ebolavirus GP Base Region

**Figure 6:**
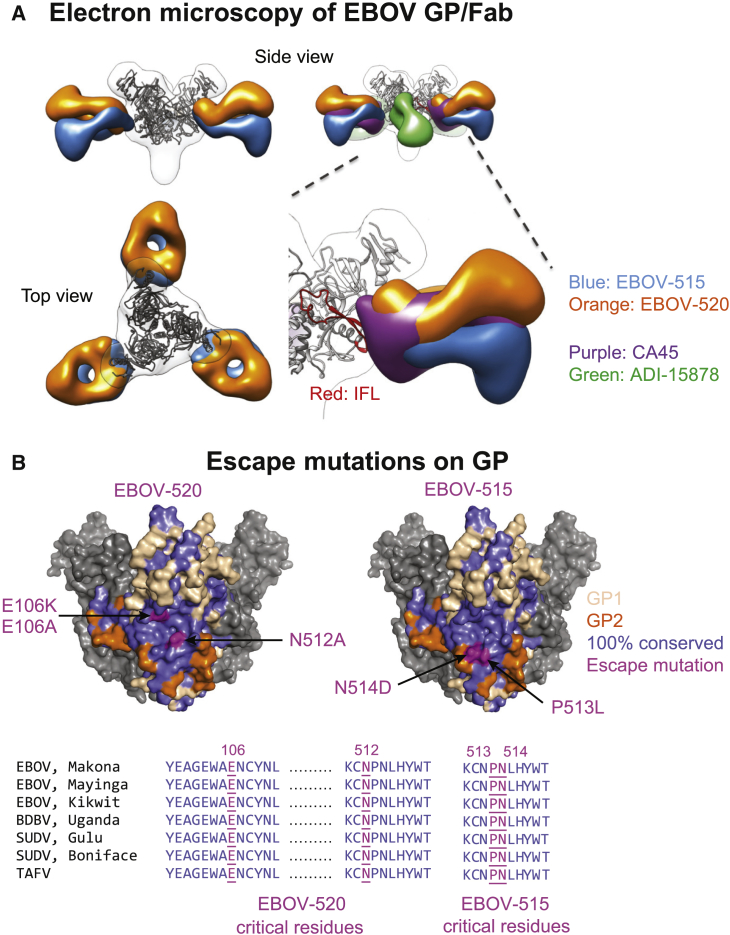
EBOV-515 and -520 Recognize Distinct Vulnerable Epitopes in the Ebolavirus GP Base Region (A) 3D reconstructions of Fab/EBOV GP ΔTM complexes. EM density for Fab of EBOV-515 (blue), or -520 (orange), or previously described CA45 (violet), or ADI-15878 (green) are superimposed to compare the angle of approach for these four GP base-reactive mAbs. A model of the EBOV GP ΔTM trimer was fitted into the EM density. (B) Escape mutations (magenta) for EBOV-515 or -520 identified by alanine-scanning mutagenesis of cell surface-displayed EBOV GP library or by sequence analysis of escape mutant viruses (top). Conservation of ebolavirus GP sequences within putative mAb epitopes (bottom). See also Figure S5.

To define the structural basis of broad neutralization by the isolated mAbs, we performed negative-stain single-particle electron microscopy (EM) studies using complexes of EBOV-515 or -520 Fab with recombinant trimeric EBOV GP ΔTM. The EM class averages obtained showed the binding of three Fab molecules on each GP trimer and confirmed recognition of the base region of GP by both mAbs (Figures 6A, S5A, and S5B). We overlaid the class averages of EBOV-515 and -520 Fab bound to GP over a class average of Fab/EBOV GP ΔTM complexes for two previously identified bNAbs: CA45 and ADI-15878 (Wec et al., 2017, Zhao et al., 2017). The structures showed that the epitope of EBOV-515 is similar to that of CA45, which recognizes the IFL region on GP2, and also GP1 below the IFL. However, EBOV-515 approaches GP with different angles than does CA45. EBOV-520 bound to a region closer to the head region of GP1, above the IFL region epitope of antibody CA45. The binding sites and approach angles of EBOV-515 and -520 differ from that of mAb ADI-15878, which binds to a non-overlapping adjacent epitope on the IFL and with a relative rotation of about 90° about the long axis (Figures 6A and S5B).

To define the epitope for the bNAbs, we used alanine scanning mutagenesis of GP and tested the binding of mAb EBOV-515 or -520 to individual GP members of a shotgun mutagenesis alanine mutation library of EBOV GP. We also generated antibody escape mutant viruses by passing infectious EBOV or rVSV/EBOV-GP in the presence of mAb and determined the GP sequence of escape variants. Consistent with the EM data, the virus escaped EBOV-515 neutralization by independent mutations at IFL residues P513L or N514D. None of the single alanine mutants affected binding of EBOV-515, likely due to its high-affinity binding mode. For EBOV-520, the N512A (GP2, IFL) and E106A (GP1 head region) mutations reduced binding to GP, and escape mutation E106K in the head region of GP1 reduced neutralizing potency (Figure 6B, top; Figures S5C and S5D). The EBOV-520 epitope is a quaternary structure located within a continuous and highly conserved region that spans the GP1 and GP2 subunits (Figure 6B, top). The residues critical for EBOV-515 or -520 binding are 100% identical among multiple ebolaviruses, including EBOV, BDBV, SUDV, and TAFV (Figure 6B, bottom), which explains the high level of neutralization breadth of these mAbs. The EBOV-520 epitope and EM analysis also suggested that, in addition to any allosteric alteration of the NPC1-C binding site, this mAb would further impede binding by full-length NPC1, since a co-crystal of GP_CL_ and NPC1 (PDB: 5JNX) (Gong et al., 2016) shows that the lumenal N-terminal domain of NPC1 is in close proximity to N512 and E106 and that EBOV-520 would sterically hinder access by NPC1.

In summary, epitope mapping studies showed that EBOV-515 and -520 recognized vulnerable epitopes in the ebolavirus GP base region. EBOV-520 appears to bind a unique highly conserved, quaternary epitope near the RBS, and therefore it represents a distinct class of potent, human mAb that could act principally by direct virus neutralization.

### EBOV-515 and -520 Mediate Protection against Heterologous SUDV or BDBV Challenge

**Figure 7:**
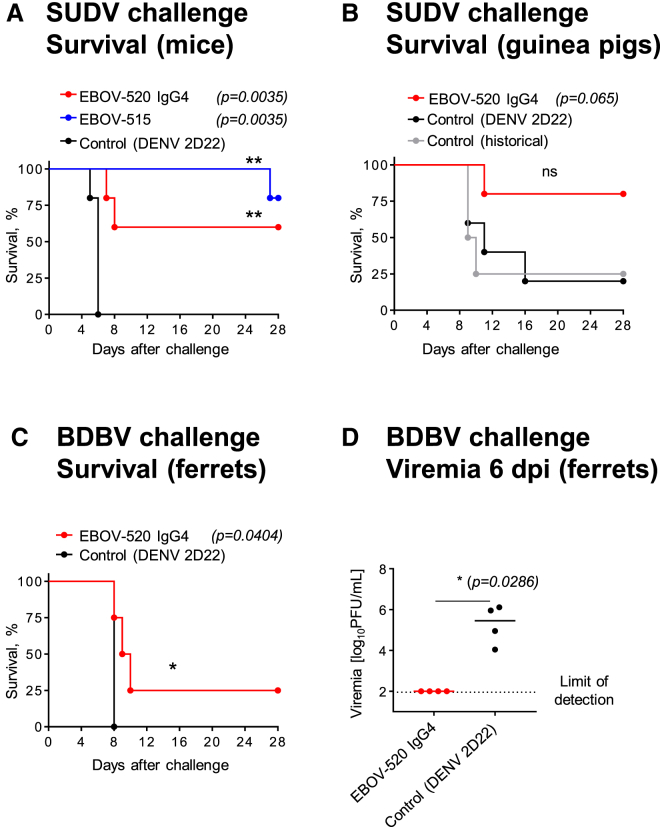
EBOV-515 and -520 Mediate Protection against Heterologous SUDV or BDBV Challenge (A) STAT1 KO mice (n = 5 per group) were inoculated with WT SUDV, treated at 1 dpi with indicated mAb, and monitored for 28 days. (B) Guinea pigs (n = 4–5 per group) were inoculated with SUDV-GA, treated on 1 and 3 dpi with indicated mAb, and monitored for 28 days. Historical controls shown included untreated animals from a separate study for comparative purposes. (C) Ferrets (n = 4 per group) were inoculated with BDBV, treated on 3 and 6 dpi with indicated mAb by i.p. injection, and monitored for 28 days. (D) A comparison of viral load in blood that was determined at 6 dpi for treated or control animals, as in (C). Median of titer for each group is shown. Data represent one experiment. Survival curves were estimated using the Kaplan Meier method and curves compared using the two-sided log rank test. Viral titers were compared using a Mann-Whitney U test. ^∗^p < 0.05 and ^∗∗^p < 0.01. See also Figures S6 and S7.

We tested the post-exposure efficiency of EBOV-515 or -520 against SUDV using a stringent STAT1-deficient (STAT1 KO) mouse challenge model (Raymond et al., 2011), in which 100% of animals in the mock-treated group succumbed to the disease by 6 dpi. A single treatment with EBOV-515 IgG1 or -520 WT IgG4 (10 mg/kg) conferred significant protection against mortality, with 80% or 60% of animals in the respective mAb treatment group surviving by 28 dpi (Figure 7A). EBOV-520 afforded partial protection against guinea pig-adapted SUDV (SUDV-GA) after challenge in a guinea pig model (Wong et al., 2015), when mAb (∼15 mg/kg) was delivered on 1 and 3 dpi (Figure 7B). We next determined efficacy of mAb EBOV-520 treatment against BDBV infection using a ferret model (Kozak et al., 2016). Ferrets were challenged with a lethal dose of BDBV and treated at 3 and 6 dpi with 18 mg of EBOV-520 or DENV 2D22 as a control by i.p. injection. All control animals became ill by 7 dpi. Two of them succumbed to the infection between observations, and two were euthanized 8 dpi as mandated by IACUC. In the EBOV-520 mAb-treated group, the male animal survived and showed no disease, while female animals became ill and were euthanized on 8–10 dpi (Figures 7C and S6). At time of the second i.p. treatment with mAb (6 dpi), all control animals developed high viremia with an infectious BDBV load that ranged from 10^4^ to 10^6^ PFU per mL of blood, and >10^7^ at 7 dpi as measured by plaque assay. In contrast, all EBOV-520-treated animals had undetectable infectious virus levels in blood on 6 dpi, and only one of three animals that succumbed showed detectable viremia on 10 dpi (Figures 7D and S6). The plaque assay, which detects infectious virus not neutralized by mAb, suggested that treatment with EBOV-520 reduced viremia. No obvious difference was observed for weight change or blood chemistry markers between the two groups (Figures S6 and S7). Given the incomplete protection observed, the physiological relevance and the efficacy of monotherapy with EBOV-520 IgG4 isotype against BDBV is uncertain, and an IgG1 form of the mAb would be preferred for future development as a therapeutic antibody. However, a significant difference in survival and infectious viral load in blood mediated by the IgG4 suggested that EBOV-520 has capacity to protect against BDBV. Together, these findings revealed that newly identified bNAbs can mediate protection against the heterologous ebolavirus infection.

## Discussion

Here we describe potent pan-ebolavirus reactive human mAbs. The work demonstrates several principles of broad protection. First, the studies identify unique binding sites for base region human mAbs that confer broad and potent activity against EBOV, BDBV, and SUDV, including engagement of a quaternary epitope spanning GP1 and GP2 subunits. Second, the work identifies bNAbs that mediate protection as monotherapy *in vivo* solely by neutralizing activity, including by a naturally occurring IgG4 antibody. Third, the EBOV-520 that binds to the GP base region reduces GP binding to the soluble NPC1-C, by an allosteric effect. Fourth, we describe pan-ebolavirus glycan cap-specific mAbs that “prime” the GP to enhance accessibility of the deep base region site of vulnerability, establishing a rational principle for development of broad mAb cocktails for ebolavirus prevention or therapy.

Two recent studies identified the IFL as a site of broad vulnerability on the ebolavirus GP for antibody recognition and reported isolation of potent macaque CA45 (Zhao et al., 2017) and two clonally related human mAbs, ADI-15878 and ADI-15742 (Wec et al., 2017). Systematic analysis of EBOV-515 and -520 extended these findings by defining new structural features of broad human antibody-mediated responses against ebolaviruses. We show that the positions and epitopes bound by EBOV-515 and -520 differ from those of ADI-15878 and ADI-15742. EBOV-520 recognizes a discontinuous epitope that spans both GP subunits with a binding pose shifted upward toward the GP head. The EBOV-515 epitope overlaps in part with that of CA45, but the mAb engages GP with different contact residues. CA45 recognized Y517, G546, and N550 residues toward the C terminus of the IF and residue R64 in the GP1 N terminus (Zhao et al., 2017). EBOV-515 recognized the P513 and N514 residues toward the N terminus of the IFL. Recognition of diverse epitopes in the base region near the IFL with differing binding poses, breadth, potency, and mechanism of action by EBOV-515, EBOV-520, CA45, ADI-15878, and ADI-15742 is reminiscent of findings for recognition of HIV Env by diverse human mAbs (Wibmer et al., 2015). Study of the epitopes in this region of GP may inform rational vaccine design against ebolaviruses.

Recent studies have emphasized an important role of Fc-mediated Ab function for protective human immunity against many viruses (Lu et al., 2018). The human neutralizing mAbs that have conferred protection to date in small animal models of EVD are of the IgG1 or IgG3 isotype, and hence likely also may function *in vivo* through the Fc region to protect. It was not clear whether neutralization alone is sufficient for protection against EVD with mAb monotherapy, or whether this activity also must be complemented by Fc-mediated function from the same mAb or another mAb in a therapeutic cocktail. In this study we showed that EBOV-520 was fully protective in mice when tested as a functionally impaired IgG1 LALA. This finding suggests that some pan-ebolavirus human mAbs can act principally or solely through neutralization to confer protection *in vivo*.

Tuning Fc-mediated effector functions of ebolavirus-neutralizing Abs is a promising strategy to enhance their activity. Here we show that the IgG1 form of EBOV-520 also can function through the Fc *in vitro*. Further studies are required to determine whether the protective effect of EBOV-520 and related clones that act principally by neutralization can be improved by tuning the Fc-mediated function up or down.

Blocking attachment of viruses to receptors on host cells is an effective antiviral strategy, but the RBS on the intact ebolavirus GP is difficult to access prior to cleavage in the endosome. Here, the findings suggest a new alternate mechanism for inhibiting GP attachment to NPC1, mediated by the base region-specific mAb EBOV-520. Binding of EBOV-520 appears to alter the conformation of the RBS by an allosteric effect that precludes proper engagement by NPC1, although the resolution of our EM studies did not allow us to determine whether a structural alteration occurred in the RBS on binding. Atomic resolution crystallography studies in the future will be required to determine the extent of this effect.

Combination therapy with a cocktail of several potent mAbs has been considered necessary for treatment of ebolavirus infections (Saphire and Aman, 2016), because it has been challenging to achieve a strong protective effect *in vivo* with monotherapy. For example, treatment with the bNAbs ADI-15742 or ADI-15878 achieved only partial protection when one considers the results from infection models for each of the three clinically relevant species (EBOV, BDBV, and SUDV) (Wec et al., 2017). Our study revealed partial protection by EBOV-520 IgG4 against BDBV and SUDV. We studied EBOV-520 *in vivo* as IgG4, since that was the original isotype isolated, but for therapy IgG1 is preferred. Direct comparison of the protective potency of EBOV-520 with that of previously reported broad mAbs would require tested of each side by side as IgG1s.

The optimal design principles for therapeutic cocktails for broad action against diverse ebolavirus species are still unclear. Ideally, one could identify a panel of mAbs that each broadly recognize all relevant ebolavirus species, but also contribute to the overall protective effect of the cocktail by complementary or synergistic activities. Here we identified two broadly reactive mAbs with synergistic activity, EBOV-437 and -442. Antibody EBOV-442 appears to be a promising candidate for inclusion in a combination therapy with EBOV-515 or -520, as a next-generation therapeutic antibody cocktail for ebolavirus treatment. The next step will be to assess the efficacy of combination therapy with a synergistic pair of these mAbs in NHP challenge studies with each of the three viruses.

In summary, we report here the isolation of potent human mAbs that recognize a unique site of broad vulnerability on the ebolavirus GP and that can mediate protection principally by neutralization. The work emphasizes important features of multifunctional response by which individual human mAbs can exploit several mechanisms for contributing to broad protective immunity. These mAbs and related clones are promising candidates for development as broadly protective pan-ebolavirus therapeutic molecules.

## STAR★Methods

### Key Resources Table

**Table:** 

REAGENT or RESOURCE	SOURCE	IDENTIFIER
**Antibodies**		

EBOV-434 (hybridoma-produced IgG1)	This study	N/A
EBOV-437 (hybridoma-produced IgG1)	This study	N/A
EBOV-442 (hybridoma-produced IgG1)	This study	N/A
EBOV-446 (hybridoma-produced IgG1)	This study	N/A
EBOV-451(hybridoma-produced IgG1)	This study	N/A
EBOV-502 (hybridoma-produced IgG1)	This study	N/A
EBOV-507 (hybridoma-produced IgG1)	This study	N/A
EBOV-508 (hybridoma-produced IgG1)	This study	N/A
EBOV-510 (hybridoma-produced IgG1)	This study	N/A
EBOV-511 (hybridoma-produced IgG4)	This study	N/A
EBOV-514 (hybridoma-produced IgG1)	This study	N/A
EBOV-515 (hybridoma-produced IgG1)	This study	N/A
EBOV-517 (hybridoma-produced IgG1)	This study	N/A
EBOV-518 (hybridoma-produced IgG3)	This study	N/A
EBOV-520 (hybridoma-produced IgG4)	This study	N/A
EBOV-524 (hybridoma-produced IgG4)	This study	N/A
BDBV289 (hybridoma-produced IgG1)	Flyak et al., 2016	N/A
BDBV317 (hybridoma-produced IgG1)	Flyak et al., 2018	N/A
BDBV223 (hybridoma-produced IgG3)	Flyak et al., 2018	N/A
4G7 (recombinant CHO-produced IgG1)	Qiu et al., 2014; this study	N/A
2G4 (recombinant CHO-produced IgG1)	Qiu et al., 2014; this study	N/A
KZ52 (recombinant CHO-produced IgG1)	(Maruyama et al., Lee et al., 2008); this study	N/A
13C6 (recombinant CHO-produced IgG1)	Qiu et al., 2014; this study	N/A
2D22 (hybridoma-produced IgG1)	Fibriansah et al., 2015	N/A
Goat anti-human IgG-HRP	Southern Biotech	Cat# 2040-05
Goat anti-human IgG-PE	Southern Biotech	Cat# 2040-09
MR72 (hybridoma-produced IgG1)	Flyak et al., 2015	N/A
MR78 (hybridoma-produced IgG1)	Flyak et al., 2015	N/A
Mouse Anti-Human IgG_1_ Hinge-AP	Southern Biotech	Cat# 9052-04
Mouse Anti-Human IgG_2_ Fc-AP	Southern Biotech	Cat# 9070-04
Mouse Anti-Human IgG3 Hinge-AP	Southern Biotech	Cat# 9210-04
Mouse Anti-Human IgG4 Fc-AP	Southern Biotech	Cat# 9200-04
EBOV-520 rIgG1 (recombinant CHO-produced)	This paper	N/A
EBOV-520 rIgG1-LALA (recombinant CHO-produced)	This paper	N/A
EBOV-520 Fab	This paper	N/A
EBOV-515 Fab	This paper	N/A
EBOV-520/Alexa Fluor 647	This paper	N/A
EBOV-515/Alexa Fluor 647	This paper	N/A
MR72/Alexa Fluor 647	This paper	N/A
MR78/Alexa Fluor 647	This paper	N/A
KZ52/Alexa Fluor 647	This paper	N/A
13C6/Alexa Fluor 647	This paper	N/A
2G4/Alexa Fluor 647	This paper	N/A
4G7/Alexa Fluor 647	This paper	N/A
BDBV289/Alexa Fluor 647	This paper	N/A
BDBV317/Alexa Fluor 647	This paper	N/A
BDBV223/Alexa Fluor 647	This paper	N/A
EBOV-442/Alexa Fluor 647	This paper	N/A
PE anti-DYKDDDDK (FLAG) Tag Antibody (clone L5)	BioLegend	Cat# 637310
Alexa Fluor 488 AffiniPure Goat Anti-Human IgG	Jackson ImmunoResearch Laboratories	Cat# 109-545-006
2G12	Polymun Scientifics	Cat# AB002
c13C6	IBT Bioservices	
Pacific Blue anti-human CD66b Antibody (clone G10F5)	BioLegend	Cat# 305112
Alexa Fluor 700 Mouse Anti-Human CD3 (clone UCHT1)	BD Biosciences	Cat# 557943
APC-Cy7 Mouse Anti-Human CD14 (clone MφP9)	BD Biosciences	Cat# 561709
PE-Cy5 Mouse Anti-Human CD107a (clone H4A3)	BD Biosciences	Cat# 555802
PE-Cy7 Mouse Anti-Human CD56 (clone B159)	BD Biosciences	Cat# 557747
APC-Cy7 Mouse Anti-Human CD16 (clone 3G8)	BD Biosciences	Cat# 557758
APC Mouse Anti-Human IFN-γ (clone B27)	BD Biosciences	Cat# 554702
PE Mouse Anti-Human MIP-1β (clone D21-1351)	BD Biosciences	Cat# 550078
Goat-anti rabbit IgG polyclonal antibody/HRP	KPL	Cat# 474-1516

**Bacterial and Virus Strains**		

Mouse-adapted EBOV /Mayinga (EBOV/M.mus-tc/COD/76/Yambuku-Mayinga)	Bray et al., 1998	GenBank: AF499101
EBOV-eGFP/Mayinga	Towner et al., 2005	N/A
Guinea pig-adapted SUDV/ Boniface (SUDV-GA)	Wong et al., 2015	GenBank: KT878488
SUDV strain Gulu	Sanchez and Rollin, 2005	GenBank: AY729654
BDBV strain 200706291 Uganda	Towner et al., 2008	GenBank: FJ217161
Chimeric EBOV/BDBV-GP	Ilinykh et al., 2016	GenBank: KU174137
Chimeric EBOV/SUDV-GP	Ilinykh et al., 2016	GenBank: KU174142
rVSV/EBOV-GP/Mayinga	Garbutt et al., 2004	N/A
E106K EBOV-eGFP, mAb EBOV-520 escape mutant	This paper	N/A
P513L EBOV-eGFP, mAb EBOV-515 escape mutant	This paper	N/A
N514D rVSV/EBOV-GP, mAb EBOV-515 escape mutant	This paper	N/A
L273P EBOV-eGFP, mAb EBOV-442 escape mutant	This paper	N/A
L273P rVSV/EBOV-GP, mAb EBOV-442 escape mutant	This paper	N/A

**Biological Samples**		

PBMCs from EVD survivor (2013-2016 EVD epidemic in Nigeria)	This paper	Donor ID #963
PBMCs from EVD survivor (2014 Boende outbreak in the DRC)	This paper	UCLADRC ES-43923
Plasma from EVD survivor (2013-2016 EVD epidemic in Nigeria)	This paper	Donor ID #963
Normal human plasma	American Red Cross	N/A

**Chemicals, Peptides, and Recombinant Proteins**		

Standard guinea pig complement	CEDARLANE Labs	Cat# CL5000
EBOV GP ΔTM (aa 1-636; Makona)	This paper	N/A
BDBV GP ΔTM (aa 1-643; 200706291 Uganda)	This paper	N/A
SUDV GP ΔTM (aa 1-637; Gulu)	This paper	N/A
MARV GP ΔTM (aa 1-648; Angola 2005)	This paper	N/A
EBOV GPΔMuc	Murin et al., 2014	N/A
EBOV GP ΔTM	IBT Bioservices	Cat# 0501-016
Thermolysin	Promega	Cat# 9PIV400
Immobilized papain	ThermoFisher	Cat# 20341
Brefeldin A	Sigma Aldrich	Cat# B7651
GolgiStop	BD Biosciences	Cat# 554724
Step-Tactin resin	QIAGEN	Cat# 30002
Alexa Fluor 647 NHS ester	ThermoFisher	Cat# A37573
Recombinant NPC1 protein, His/FLAG-tagged	Creative BioMart	Cat# NPC1-1339H
FluoSpheres NeutrAvidin-Labeled Microspheres	ThermoFisher	Cat# F-8776
EZ-Link Sulfo-NHS-LC-LC-Biotin	ThermoFisher	Cat# 21338
1-Step Ultra TMB-ELISA	ThermoFisher	Cat# 34029
Freestyle 293 expression medium	ThermoFisher	Cat# 12338002
ExpiCHO Expression Medium	ThermoFisher	Cat# A2910001
Fetal Bovine Serum, ultra-low IgG	ThermoFisher	Cat# 16250078
ClonaCell-HY Medium E	Stem Cell Technologies	Cat# 03805
ClonaCell-HY Medium A	Stem Cell Technologies	Cat# 03801

**Critical Commercial Assays**		

RosetteSep Human NK Cell Enrichment Cocktail	Stem Cell Technologies	Cat# 15025
Diagnostic Profile Reagent Rotor Package	Abaxis	Cat# 500-0038

**Deposited Data**		

EBOV-515 Fab complex with EBOV GP ΔTM	This paper	EMD-7956
EBOV-520 Fab complex with EBOV GP ΔTM	This paper	EMD-7955

**Experimental Models: Cell Lines**		

Human: Jurkat, clone E6-1	ATCC	ATCC: TIB-152
Human: Jurkat-EBOV GP (Makona)	Davis and Ahmed, personal communication	N/A
Mouse: NIH 3T3-hCD40-hIL21-hBAFF	D. Bhattacharya	N/A
Mouse-human HMAA 2.5 myeloma cell line	L. Cavacini	N/A
Hamster: ExpiCHO-S	ThermoFisher Scientific	Cat# A29127
Human: FreeStyle 293F	ThermoFisher Scientific	Cat# R79007
Human: THP-1 monocytes	ATCC	ATCC: TIB-202
Human: EBOV GPkik-293FS EGFP CCR5-SNAP	J. Lewis	N/A
Monkey: Vero-E6	ATCC	ATCC: CRL-1586
*Drosophila*: Schneider 2	ThermoFisher Scientific	Cat# R69007
EBOV-434 hybridoma clone	This study	N/A
EBOV-437 hybridoma clone	This study	N/A
EBOV-442 hybridoma clone	This study	N/A
EBOV-446 hybridoma clone	This study	N/A
EBOV-451 hybridoma clone	This study	N/A
EBOV-502 hybridoma clone	This study	N/A
EBOV-507 hybridoma clone	This study	N/A
EBOV-508 hybridoma clone	This study	N/A
EBOV-510 hybridoma clone	This study	N/A
EBOV-511 hybridoma clone	This study	N/A
EBOV-514 hybridoma clone	This study	N/A
EBOV-515 hybridoma clone	This study	N/A
EBOV-517 hybridoma clone	This study	N/A
EBOV-518 hybridoma clone	This study	N/A
EBOV-520 hybridoma clone	This study	N/A
EBOV-524 hybridoma clone	This study	N/A

**Experimental Models: Organisms/Strains**		

Mouse: BALB/cJ	The Jackson Laboratory	N/A
Mouse: 129S6/SvEv-Stat1^tm1Rds^ (STAT1 KO)	Taconic Biosciences	N/A
Guinea pig: Hartley	Charles River Laboratories	N/A
Ferret: Outbred *Mustela putorius furo*	Marshall BioResources	N/A

**Recombinant DNA**		

Plasmid: EBOV GP ΔTM (aa 1-636; Makona)	This paper	N/A
Plasmid: BDBV GP ΔTM (aa 1-643; 200706291 Uganda)	This paper	N/A
Plasmid: SUDV GP ΔTM (aa 1-637; Gulu)	This paper	N/A
Plasmid: MARV GP ΔTM (aa 1-648; Angola 2005)	This paper	N/A
Plasmid: EBOV-520 rIgG1 heavy chain	This paper	N/A
Plasmid: EBOV-520 light chain	This paper	N/A
Plasmid: EBOV-520 rIgG1-LALA heavy chain	This paper	N/A
Plasmid: EBOV-520 Fab heavy chain	This paper	N/A

**Software and Algorithms**		

GraphPad Prism 7.2	GraphPad Software, Inc.	https://www.graphpad.com
FlowJo version 10	Tree Star Inc.	https://www.flowjo.com/solutions/flowjo/downloads
ImMunoGeneTics database	Giudicelli and Lefranc, 2011	http://www.imgt.org/
ForeCyt Standard 6.2 (R1)	Intellicyt	https://intellicyt.com/products/software/
MATLAB (r2015a)	MathWorks, Inc.	https://www.mathworks.com/products/matlab.html
DoGpicker	Voss et al., 2009	http://emg.nysbc.org/redmine/projects/software/wiki/DoGpicker
Appion	Lander et al., 2009	http://emg.nysbc.org/redmine/projects/appion/wiki/Appion_Home
Pymol	Schrödinger	https://www.pymol.org/2//

**Other**		

VetScan VS2 Chemistry Analyzer	Abaxis	N/A
iQue Screener Plus flow cytometer	Intellicyt	N/A
BD LSR2 (3-laser) flow cytometer	BD Biosciences	N/A
ECM 2001 Electro Cell Manipulator	BTX	N/A
ÄKTA pure chromatography system	GE Healthcare	N/A
Tecnai Spirit electron microscope with TemCam F416 4k x 4k CCD	Zhao et al., 2017	N/A
Synergy H1 microplate reader	BioTek	N/A
Synergy 2 microplate reader	BioTek	N/A
EL406 washer dispenser	BioTek	N/A
Biostack microplate stacker	BioTek	N/A
StrepTrap HP	GE Healthcare	Cat# 28-9075-48
HiTrap Protein G High Performance	GE Healthcare	Cat# 17-0404-01
HiTrap MabSelect SuRe 5 mL column	GE Healthcare	Cat# 11-0034-93
Zeba Spin Desalting Columns, 7K MWCO, 0.5 mL	ThermoFisher	Cat# PI-89883
Superdex 200 Increase 10/300 GL column	GE Healthcare	N/A

### Contact for Reagent and Resource Sharing

Further information and requests for resources and reagents should be directed to and will be fulfilled by the Lead Contact, James E. Crowe, Jr. (james.crowe@Vanderbilt.Edu). Materials described in this paper are available for distribution under the Uniform Biological Material Transfer Agreement, a master agreement that was developed by the NIH to simplify transfers of biological research materials.

### Experimental Model and Subject Details

#### Human samples

Human PBMCs were obtained from survivors of the 2014 EVD epidemic in Nigeria or of the 2014 Boende outbreak in the Democratic Republic of the Congo (DRC) (unpublished). A male human survivor of the 2014 EVD outbreak in Nigeria was age 31 y when infected and age 32 when PBMCs were collected. A male human survivor of the 2014 Boende outbreak in the DRC was age 36 y when infected and age 37 y when PBMCs were collected. PBMCs were collected well after the illness had resolved, following informed consent. At time of blood collection, plasma samples were tested by RT-PCR and found to be negative for the presence of viral RNA. The studies were approved by the Institutional Review Boards of Vanderbilt University Medical Center, the UCLA Fielding School of Public Health and the Kinshasa School of Public Health (DRC).

#### Cell lines

Vero-E6 (monkey, female origin), THP-1 (human, male origin), and Jurkat (human, male origin) cell lines were obtained from the American Type Culture Collection. Vero-E6 cells were cultured in Minimal Essential Medium (MEM) (ThermoFisher Scientific) supplemented with 10% fetal bovine serum (HyClone) and 1% penicillin-streptomycin at 5% CO_2_, 37°C. THP-1 and Jurkat cells were cultured in RPMI 1640 (GIBCO) medium supplemented with 10% heat-inactivated fetal bovine serum (GIBCO), 1% GlutaMax (GIBCO), and 1% penicillin-streptomycin (GIBCO) at 37°C in 5% CO_2_. The HMAA 2.5 non-secreting mouse-human heteromyeloma cell line (sex information is not available) was a kind gift from L. Cavacini and was cultured as described previously (Yu et al., 2008). A 293F cell line (human, female origin) stably-transfected to express SNAP-tagged EBOV GP was described recently (Domi et al., 2018). ExpiCHO (hamster, female origin) and FreeStyle 293F (human, female origin) cell lines were purchased from ThermoFisher Scientific and cultured according to the manufacturer’s protocol. The Jurkat-EBOV GP cell line stably expressing EBOV GP Makona on the surface (Davis and Ahmed, personal communication) was a kind gift from Carl Davis (Emory University, Atlanta, GA). An NIH 3T3 engineered fibroblast line (mouse, male origin) constitutively expressing cell-surface human CD154 (CD40 ligand), secreted human B cell activating factor (BAFF) and human IL-21 was kindly provided by Dr. Deepta Bhattacharya (Washington University in St. Louis, MO). All cell lines were tested on a monthly basis for Mycoplasma and found to be negative in all cases.

#### Viruses

The authentic EBOV-eGFP, mouse-adapted EBOV Mayinga (EBOV-MA, GenBank: AF49101), guinea pig-adapted SUDV (SUDV-GA, GenBank: KT878488), SUDV strain Gulu, and BDBV strain 200706291 Uganda viruses were described previously (Bray et al., 1998, Sanchez and Rollin, 2005, Towner et al., 2005, Towner et al., 2008, Wong et al., 2015). The chimeric infectious EBOV/BDBV-GP and EBOV/SUDV-GP viruses expressing eGFP were obtained by replacing the gene encoding EBOV GP with that of BDBV (GenBank: KU174137) or SUDV (GenBank: KU174142), respectively (Ilinykh et al., 2016), and passaged two times in Vero-E6 cell culture monolayers. Recombinant chimeric vesicular stomatitis virus in which the G protein was replaced with EBOV GP (rVSV/EBOV-GP) were provided by Heinz Feldmann (Rocky Mountain Laboratories, NIH, Hamilton, MT) (Garbutt et al., 2004).

#### Mouse models

Seven- to eight-week old female BALB/c mice were obtained from the Jackson Laboratory, and 7-8 week-old 129S6/SvEv-Stat1^tm1Rds^ mice (STAT1 KO) were obtained from Taconic Biosciences. Mice were housed in microisolator cages and provided food and water *ad libitum*. Challenge studies were conducted under maximum containment in an animal biosafety level 4 (ABSL-4) facility of the Galveston National Laboratory, UTMB.

#### Guinea pig model

Five- to six-week-old female Hartley guinea pigs were obtained from the Charles River Laboratories. Animals were housed and challenged under maximum containment in ABSL-4 facility of the Galveston National Laboratory, UTMB.

#### Ferret model

Six-month-old male and female ferrets (*Mustela putorius furo*) were obtained from Marshall BioResources. Animals were housed and challenged under maximum containment in ABSL-4 facility of the Galveston National Laboratory, UTMB.

The animal protocols for testing of mAbs in mice, guinea pigs and ferrets were approved by the Institutional Animal Care and Use Committee of the University of Texas Medical Branch (UTMB) in compliance with the Animal Welfare Act and other applicable federal statutes and regulations relating to animals and experiments involving animals.

### Method Details

#### Mouse challenge with EBOV

Groups of 7-8-week-old female BALB/c mice (n = 5 per group) housed in microisolator cages were inoculated with 1,000 PFU of the EBOV-MA by the intraperitoneal (i.p.) route. Mice were treated i.p. with 100 μg (∼5 mg/kg) of individual mAb per mouse on 1 dpi. Human mAb DENV 2D22 (specific to an unrelated target, dengue virus) served as negative control. Mice were monitored twice daily from day 0 to 14 dpi for illness, survival, and weight loss, followed by once daily monitoring from 15 dpi to the end of the study at 28 dpi. The extent of disease was scored using the following parameters: dyspnea (possible scores 0–5), recumbence (0–5), unresponsiveness (0–5), and bleeding/hemorrhage (0–5). Moribund mice were euthanized as per the IACUC-approved protocol. All mice were euthanized on day 28 after EBOV challenge.

#### Mouse challenge with SUDV

Groups of 7-8-week-old STAT1 KO mice (n = 5 per group) were challenged i.p. with 1,000 PFU *wt* SUDV (Gulu). Animals were treated i.p. with 200 μg (∼10 mg/kg) of EBOV-specific or control mAb DENV 2D22 per mouse on 1 dpi and were monitored as above.

#### Guinea pig challenge with SUDV

Groups of 5- to 6-week-old Hartley guinea pigs (n = 5/group) were injected i.p. with 1,000 PFU of SUDV-GA (guinea pig adapted strain Boniface) (Wong et al., 2015). mAb EBOV-520 was delivered by i.p. route at indicated time points and doses. Control groups were treated with mAb DENV 2D22 or left untreated. Animals were monitored for the illness, survival, and weight loss. All animals were euthanized at 28 dpi.

#### Ferret challenge with BDBV

Groups of 6-month-old male and female animals were challenged intramuscularly with 1,000 PFU of BDBV, as described previously (Kozak et al., 2016). Animals were treated by i.p. route with 18 mg of mAb EBOV-520 or the control mAb DENV 2D22 on day 3, and the same dose of the mAb on day 6 after challenge. The disease scores were assessed as follows: healthy, 1; developing clinical disease, 2; advanced disease, 3; moribund, 4. Blood was collected from surviving animals on 0, 3, 6, 9, 14, 21, and 28 dpi to assess virus titers. Ferrets were monitored for 28 days after virus inoculation and then euthanized.

#### Generation of human B cell hybridomas producing mAbs

PBMCs from heparinized blood were isolated with Ficoll-Histopaque by density gradient centrifugation. The cells were cryopreserved in the vapor phase of liquid nitrogen until use. Human B cell hybridomas were generated as described previously (Yu et al., 2008) with some modifications. Briefly, previously cryopreserved samples were thawed and expanded on irradiated NIH 3T3 cells that had been engineered to express human IL-21, CD40L, and BAFF in medium A (STEMCELL Technologies) supplemented with CpG, a Chk2 inhibitor (Sigma), and cyclosporine A (Sigma). After 7 days, supernatants from each well of the 384-well culture plates were assessed by ELISA for reactivity against various ebolavirus proteins using enzyme-linked immunosorbent assays (ELISAs), as described below. The next day, cells from wells with supernatants reacting with antigen in an ELISA were fused with HMMA2.5 myeloma cells using an established electrofusion technique (Yu et al., 2008). After the fusion reaction, hybridoma lines were cultured in ClonaCell-HY Medium E (STEMCELL Technologies) supplemented with HAT Media Supplement (Sigma) in 384-well plates for 18 days before screening of supernatants for antibody production. Hybridoma cell lines producing ebolavirus GP-reactive antibodies were cloned biologically by single-cell fluorescence-activated cell sorting. Hybridomas were expanded in Medium E until 50% confluent in 75-cm^2^ flasks (Corning).

#### mAb isotype and gene sequence analysis

The isotype and subclass of secreted antibodies were determined using murine anti-human IgG1, IgG2, IgG3 or IgG4 mouse antibodies conjugated with alkaline phosphatase (Southern Biotech). Antibody heavy- and light-chain variable region genes were sequenced from hybridoma lines that had been cloned biologically from flow cytometry. Briefly, total RNA was extracted using the RNeasy Mini kit (QIAGEN) and reverse-transcriptase PCR (RT-PCR) amplification of the antibody gene cDNAs was performed using the PrimeScript One Step RT-PCR kit (CLONTECH) according to the manufacturer’s protocols with gene-specific primers (Thornburg et al., 2016). The thermal cycling conditions were as follows: 50°C for 30 min, 94°C for 2 min, 40 cycles of (94°C for 30 s, 58°C for 30 s and 72°C for 1 min). PCR products were purified using Agencourt AMPure XP magnetic beads (Beckman Coulter) and sequenced directly using an ABI3700 automated DNA sequencer. The identities of gene segments and mutations from germlines were determined by alignment using the ImMunoGeneTics database (Giudicelli and Lefranc, 2011).

#### mAb production and purification

Hybridoma cells secreting GP-reactive mAbs were grown in serum-free medium (Hybridoma-SFM, Life Technologies). MAbs were purified from filtered culture supernatants by fast protein liquid chromatography (FPLC) on an ÄKTA instrument using HiTrap MabSelect Sure or HiTrap Protein G columns (GE Healthcare). Purified mAbs were buffer exchanged into PBS, filtered using sterile 0.45-μm pore size filter devices (Millipore), concentrated, and stored in aliquots at −80°C until use.

For recombinant mAb production, cDNA encoding the genes of heavy and light chains were cloned into DNA plasmid expression vectors encoding IgG (IgG1, IgG3, Ig4, or IgG1-LALA) - or Fab- heavy chain (McLean et al., 2000) and transformed into *E. coli* cells. mAb proteins were produced following transiently transfection of FreeStyle 293F or ExpiCHO cells following the manufacturer’s protocol and were purified as described above.

#### GP expression and purification

The ectodomains of EBOV GP ΔTM (residues 1-636; strain Makona; GenBank: KM233070), BDBV GP ΔTM (residues 1-643; strain 200706291 Uganda; GenBank: NC_014373), SUDV GP ΔTM (residues 1-637; strain Gulu; GenBank: NC_006432), and MARV GP ΔTM (residues 1-648; strain Angola2005; GenBank: DQ447653) were expressed transiently in Expi293F cells with a C-terminal strep II tag using the pcDNA3 plasmid vector. Secreted proteins were purified using 5 mL StrepTrap HP column (GE Healthcare) following the manufacturer’s protocol, and then purified further and buffer exchanged into PBS using Supedex200 (GE Healthcare) size exclusion chromatography. Formation of EBOV GP ΔTM trimer was confirmed by negative stain EM. For some experiments, we used EBOV GP that was produced in *Drosophila* Schneider 2 (S2) cells. Briefly, recombinant ectodomain of EBOV GP ΔTM in modified pMTpuro vector was transfected into S2 cells followed by stable selection of transfected cells with 6 μg/mL puromycin. GP ectodomain expression was induced with 0.5 mM CuSO_4_ for 4 days. Protein was purified using Strep-Tactin resin (QIAGEN) via an engineered strep II tag and purified further by Superdex 200 (S200) column chromatography. Purity of recombinant GP was confirmed by SDS-PAGE.

#### ELISA binding assays

Wells of microtiter plates were coated with purified, recombinant EBOV, BDBV, SUDV, or MARV GP ΔTM and incubated at 4°C overnight. Plates were blocked with 2% non-fat dry milk and 2% normal goat serum in DPBS containing 0.05% Tween-20 (DPBS-T) for 1 hr. For mAb screening assays, hybridoma culture supernatants were diluted in blocking buffer 1:5, added to the wells, and incubated for 1 hr at ambient temperature. The bound antibodies were detected using goat anti-human IgG conjugated with HRP (Southern Biotech) and TMB substrate (ThermoFisher). Color development was monitored, 1N hydrochloric acid was added to stop the reaction, and the absorbance was measured at 450 nm using a spectrophotometer (Biotek).

For dose-response and cross-reactivity assays, serial dilutions of plasma or purified mAbs were applied to the wells in triplicate or quadruplicate, as detailed above. EC_50_ values for mAb binding were determined using Prism 7.2 software (GraphPad) after log transformation of antibody concentration using sigmoidal dose-response nonlinear regression analysis. Similarly, a non-linear regression analysis was performed on the resulting curves to calculate plasma dilution that yielded a half-maximum O.D. 450 nm value. Antibody titer in plasma was expressed as the inverse of plasma dilution.

#### Cell surface displayed GP mAb binding

Jurkat-EBOV GP cells were washed with the incubation buffer containing DPBS, 2% of heat-inactivated FBS and 2 mM EDTA (pH 8.0) by centrifugation at 400 x *g* for 5 min at room temperature. For antibody staining, ∼5 × 10^4^ cells were added per each well of V-bottom 96-well plate (Corning) in 5 μL of the incubation buffer. Serial dilutions of antibody were added to the cells in triplicate or quadruplicate for total volume of 50 μL per well, followed by 1 hr incubation at room temperature, or 4°C in some experiments. Unbound antibody was removed by washing with 200 μL of the incubation buffer as described above, and cells were incubated with phycoerythrin (PE)-labeled secondary goat anti-human antibodies (Southern Biotech) for 30 min at 4°C. In some experiments, cells were fixed with 4% PFA in DPBS before staining with secondary antibodies. Staining of cells was measured by flow cytometric analysis using an Intellicyt iQue high throughput cytometer (Intellicyt), or an LSRII flow cytometer (BD Biosciences). Data for up to 20,000 events were acquired, and data were analyzed with ForeCyt (Intellicyt) or FlowJo (Tree Star) software. Dead cells were excluded from the analysis on the basis of forward and side scatter gate for viable cell population. Binding to untransduced Jurkat cells, or binding of dengue antigen-specific mAb DENV 2D22 served as negative controls for most experiments.

In some experiments, binding to cell surface displayed GP was assessed with mAbs that were directly fluorescently-labeled. Briefly, mAbs were labeled with Alexa Fluor 647 NHS ester (ThermoFisher) by following the manufacturer’s protocol. Labeled mAbs were purified further and buffer exchanged into the PBS using desalting Zeba columns (ThermoFisher) and stored at 4°C with 0.1% bovine serum albumin (Sigma) and 0.01% sodium azide.

To assess binding of mAbs to Jurkat-EBOV GP_CL_, Jurkat-EBOV GP cells were counted and treated with 0.5 mg/mL of thermolysin (Promega) in PBS for 20 min at 37°C. Cell staining and flow cytometric analysis was performed as described above. Binding to untransfected Jurkat or uncleaved Jurkat-EBOV GP served as controls.

#### Cell surface displayed GP mAb competition-binding

Jurkat-EBOV GP or Jurkat-EBOV_CL_ cells were pre-incubated with a saturating concentration (typically 20 μg/mL) of the first unlabeled mAb at room temperature for 30 min, followed by addition of the second fluorescently-labeled mAb (typically 5 μg/mL) and incubated for an additional 30 min. The second mAb was added after the first mAb and without washing of cells to minimize a dissociation of the first mAb from cell surface GP during a prolonged incubation. Cells were washed, fixed with PFA, and cell staining was analyzed using an Intellicyt iQue flow cytometer as detailed above. Background values were determined from binding of the second labeled mAbs to untransfected Jurkat. Results are expressed as the percent of binding in the presence of competitor mAb relative to primary mAb-only control (maximal binding) minus background. The antibodies were considered competing if the presence of first antibody reduced the signal of the second antibody to less than 30% of its maximal binding or non-competing if the signal was greater than 70%. A level of 30%–70% was considered intermediate competition. Of note, mAbs from the GP base region competitor epitope group revealed much stronger binding to Jurkat-EBOV GP_CL_ than to Jurkat-EBOV GP cells. This finding was revealed by nearly complete cross-blocking capacity of these mAbs on Jurkat-EBOV GP_CL_ when compared to those determined for Jurkat-EBOV GP cells (Figure 4A, B).

#### Cell surface displayed GP cleavage inhibition

Jurkat-EBOV GP cells were pre-incubated with serial dilutions of mAbs in PBS for 20 min at room temperature, then incubated with thermolysin for 20 min at 37°C. The reaction was stopped by addition of the incubation buffer as described above. Washed cells were incubated with 5 μg/mL of fluorescently-labeled RBS-specific mAb MR78 at 4°C for 60 min. Stained cells were washed, fixed, and analyzed by flow cytometry using Intellicyt iQue. Cells were gated for the viable population. Background staining was determined from binding of the labeled mAb MR78 to Jurkat-EBOV GP (uncleaved) cells. Results are expressed as the percent of RBS exposure in the presence of tested mAb relative to labeled MR78 mAb-only control (maximal binding to Jurkat-EBOV GP_CL_) minus background.

#### Cell surface displayed GP_CL_ soluble NPC1-C binding inhibition

Jurkat-EBOV GP_CL_ cells were prepared as detailed above and resuspended in the incubation buffer. Approximately 5 × 10^4^ cells per well in V-bottom 96-well plate were incubated with serial 3-fold dilutions of mAbs in a total volume of 50 μL at ambient temperature for 30 min, followed by washing and incubation with pre-titrated concentration (typically 50 μg/mL) of soluble, FLAG epitope-tagged, recombinant NPC1- C protein (Creative BioMart). Cells were washed, incubated with PE-labeled secondary mouse anti-FLAG tag antibody (BioLegend) for 2 hr at 4°C, fixed with PFA, and then analyzed by flow cytometry using LSRII cytometer equipped with 535 nm green laser. Cells were gated for the viable population. Results are expressed as the percent of NPC1-C binding inhibition in the presence of tested mAb relative to NPC1-only control (maximal binding to Jurkat-EBOV GP_CL_) minus background.

#### Cooperative binding to cell surface displayed GP

The cell surface display assay was based on principles from previously described enhanced binding ELISA assay (Howell et al., 2017). Briefly, Jurkat-EBOV GP cells were incubated with a mixture of individual unlabeled glycan cap-specific mAbs at a saturating concentration (10 μg/mL) and respective dilution of fluorescently-labeled mAbs EBOV-515 or −520. Cells were washed, and antibody binding was analyzed by flow cytometry using Intellicyt iQue.

#### Epitope mapping using an EBOV GP alanine-scan mutation library

Epitope mapping was carried out as described previously (Davidson et al., 2015). Comprehensive high-throughput alanine scanning (‘shotgun mutagenesis’) was carried out on an expression construct for EBOV GP lacking the mucin-like domain (residues 311-461) (based on the Yambuku-Mayinga variant GP sequence), mutagenizing GP residues 33-310 and 462-676 to create a library of clones, each representing an individual point mutant. Residues were changed to alanine (with alanine residues changed to serine). The resulting library, covering 492 of 493 (99.9%) of target residues, was arrayed into 384-well plates, one mutant per well, then transfected into HEK293T cells and allowed to express for 22 hr. Cells, unfixed or fixed in 4% paraformaldehyde, were incubated with primary antibody and then with an Alexa Fluor 488-conjugated secondary antibody (Jackson ImmunoResearch Laboratories). After washing, cellular fluorescence was detected using the Intellicyt high throughput flow cytometer (Intellicyt). mAb reactivity against each mutant EBOV GP clone was calculated relative to wild-type EBOV GP reactivity by subtracting the signal from mock-transfected controls and normalizing to the signal from wild-type GP-transfected controls. Mutated residues within clones were identified as critical to the mAb epitope if they did not support reactivity of the test mAb but did support reactivity of other control EBOV mAbs. This counter-screen strategy facilitated the exclusion of GP mutants that were misfolded locally or that exhibited an expression defect. The detailed algorithms used to interpret shotgun mutagenesis data were described previously (Davidson and Doranz, 2014).

#### Generation of virus neutralization escape mutants

To generate escape mutants for EBOV-520 and −442 mAbs, 100 PFU of EBOV-eGFP were combined with 2-fold dilutions of the respective mAb starting at 200 μg/mL in U-bottom 96-well plates and incubated for 1 hr at 37°C. Mixtures were placed on Vero-E6 cell monolayer cultures in 96-well plates and incubated for 1 hr. Supernatants were removed, freshly-diluted mAb was added at the same concentrations in 200 μL of MEM supplemented with 2% FBS, and plates were incubated for 7 days at 37°C. Viruses that replicated in the presence of the highest concentrations of mAb, as determined by monitoring eGFP fluorescence by microscopy, were collected. 20 μL aliquots were incubated with 2-fold dilutions of mAbs starting at 200 μg/mL, and viruses were propagated in the presence of mAbs as described above. The procedure was repeated once more with mAb dilutions starting at 400 μg/mL. Viruses that replicated at the highest mAb concentrations were amplified in Vero-E6 cell culture monolayers in 24-well plates in the presence of mAbs at 200 μg/mL for 7 days. Cells were used for isolation of RNA using TRIzol reagent, and cDNA copies of viral RNA encoding GP were amplified by RT-PCR and sequenced. To determine susceptibility of the isolated escape mutants to mAbs, 100 PFU of the viruses in MEM supplemented with 2% FBS in triplicate were combined in U-bottom 96-well plates with 8 to 12 two-fold dilutions of mAb, starting at 200 μg/mL, in total volumes of 50 μL, and incubated for 1 hr at 37°C. The virus/antibody mixtures then were added in triplicate to Vero-E6 cell culture monolayers in 96-well plates, incubated for 1 hr at 37°C, washed with MEM, overlaid with 200 μL of MEM containing 2% FBS and 0.8% methylcellulose, and incubated for 48 hr at 37°C. Plates were fixed with 10% phosphate-buffered formalin (Fisher). Plaques were counted using a fluorescence microscopy.

To generate EBOV-515 escape mutants, aliquots containing 100 PFU of rVSV/EBOV-GP virus were pre-incubated with serial 2-fold dilutions starting from 200 μg/mL of mAb for 1 hr at 37°C and inoculated into 96-well plate Vero-E6 cell monolayers. After 48 hr, virus samples were harvested and titrated. Virus-positive samples from the highest mAb concentration were selected for the next passage. After seven passages, a 200 PFU virus aliquot was pre-incubated with mAb EBOV-515 and inoculated into a 24-well plate Vero-E6 cell monolayer culture. After 72 hr, the infected cell monolayer was solubilized in TRIzol (Ambion, Life Technologies) and subjected to total RNA isolation, RT-PCR and sequencing of EBOV GP.

#### Neutralization assays

Antibody neutralization assays were performed in a high-throughput or plaque reduction format using the recombinant EBOV-eGFP, rVSV/EBOV-GP, or chimeric EBOV viruses in which GP was replaced with its counterpart from BDBV or SUDV as described previously (Ilinykh et al., 2016, Towner et al., 2005). For the assays with thermolysin-cleaved virus, rVSV/EBOV-GP virus was propagated in Vero-E6 cells. At 48 hr after infection, virus suspension was harvested and clarified from cell debris by centrifugation for 10 min at 10,000 x *g*. Next, the supernatant was ultracentrifuged through a 25% sucrose cushion for 2 hr at 175,000 x *g* at 4°C. Pelleted virus was resuspended in thermolysin digestion buffer (50 mM Tris, 0.5 mM CaCl_2_ pH 8.0) and divided into 2 aliquots: one aliquot was treated with 0.5 mg/mL of thermolysin (Promega), another one – with equal volume of thermolysin digestion buffer (mock-treated virus) for 40 min at 37°C. The reactions were stopped by addition of EDTA up to the final concentration 10 mM. Virus samples were re-pelleted through a 25% sucrose cushion and were washed by ultracentrifugation in buffer containing 10 mM Tris (pH 8.0) and 0.1 M NaCl for 1 hr at 175,000 x *g* at 4°C. Virus pellets were resuspended in the same buffer, incubated with serial mAb dilutions for 1 hr at 37°C, or mock-incubated, and titrated by applying to Vero-E6 cell culture monolayers in triplicate.

#### Antibody-mediated cellular phagocytosis by human monocytes (ADCP)

Recombinant EBOV GP ΔTM (IBT Bioservices) was biotinylated and coupled to Alexa Fluor 488 Neutravidin beads (Life Technologies). Antibodies were diluted to 5μg/ml in cell culture medium and incubated with beads for 2 hr at 37°C. THP-1 monocytes (ATCC) were added at 2.5 × 10^4^ cells per well and incubated for ∼18 hr at 37°C. Cells were fixed with 4% paraformaldehyde and analyzed on a BD LSRII flow cytometer, and a phagocytic score was determined using the percentage of FITC^+^ cells and the median fluorescence intensity of the FITC^+^ cells. The glycan cap-specific mAb c13C6 (IBT Bioservices) was used as a positive control, and the HIV-specific mAb 2G12 (Polymun Scientifics) was used as a negative control.

#### Antibody-mediated neutrophil phagocytosis (ADNP)

Recombinant EBOV GP ΔTM (IBT Bioservices) was biotinylated and coupled to Alexa Fluor 488 Neutravidin beads (Life Technologies). Antibodies were diluted to 5 μg/mL in cell culture medium and incubated with beads for 2 hr at 37°C. White blood cells were isolated from donor peripheral blood by lysis of red blood cells, followed by three washes with PBS. Cells were added at a concentration of 5.0 × 10^4^ cells/well and incubated for 1 hr at 37°C. Cells were stained with CD66b (Pacific Blue, Clone G10F5; BioLegend), CD3 (Alexa 700, Clone UCHT1; BD Biosciences), and CD14 (APC-Cy7, Clone MφP9; BD Biosciences), and fixed with 4% paraformaldehyde, and analyzed by flow cytometry on a BD LSR II flow cytometer. Neutrophils were defined as SSC-A^high^ CD66b^+^, CD3^-^, CD14^-^. A phagocytic score was determined using the percentage of FITC^+^ cells and the median fluorescence intensity of the FITC^+^ cells. The glycan cap-specific mAb c13C6 (IBT Bioservices) was used as a positive control, and the HIV-specific mAb 2G12 (Polymun Scientifics) was used as a negative control.

#### Antibody-dependent NK cell degranulation

Recombinant EBOV GP ΔTM (IBT Bioservices) was coated onto a MaxiSorp 96 well plates (Nunc) at 300 ng/well at 4°C for 18 hr. Wells were washed three times with PBS and blocked with 5% bovine serum albumin in PBS. Antibodies were diluted to 5 μg/mL in PBS, and added to the plates, and were incubated for an additional 2 hr at 37°C. Unbound antibodies were removed by washing three times with PBS, and human NK cells freshly isolated from peripheral blood of human donors by negative selection (Stem Cell Technologies, Canada) were added at 5 × 10^4^ cells/well in the presence of 4 μg/mL brefeldin A (Sigma Aldrich) and 5 μg/mL GolgiStop (Life Technologies) and anti-CD107a antibody (PE-Cy5, Clone H4A3, BD Biosciences). Plates were incubated for 5 hr at 37°C. Cells were stained for NK cell markers (CD56 PE-Cy7, clone B159, BD Biosciences; CD16 APC-Cy7, clone 3G8, BD Biosciences; CD3 Alexa Fluor700, clone UCHT1, BD Biosciences), followed by fixation and permeabilization with Fix and Perm (Life Technologies) according to the manufacturer’s instructions to stain for intracellular IFNγ (APC, Clone B27, BD Biosciences) and MIP-1β (PE, Clone D21-1351, BD Biosciences). Cells were analyzed on a BD LSRII flow cytometer. The glycan cap-specific mAb c13C6 (IBT Bioservices) was used as a positive control, and the HIV-specific mAb 2G12 (Polymun Scientifics, Austria) was used as a negative control.

#### Antibody-mediated complement deposition (ADCD)

Recombinant EBOV GP (IBT Bioservices) was biotinylated and coupled to red fluorescent Neutravidin beads (Life Technologies). Antibodies were diluted to 5μg/mL in RPMI-1640, and incubated with GP-coated beads for 2 hr at 37°C. Freshly reconstituted guinea pig complement (Cedarlane Labs) was diluted in veronal buffer with 0.1% fish gelatin (Boston Bioproducts), added to the antibody-bead complexes, and incubated for 20 min at 37°C. Beads were washed twice with phosphate buffered saline containing 15 mM EDTA, and stained with an anti-guinea pig C3 antibody conjugated to FITC (MP Biomedicals) for 15 min at ambient temperature. Beads were washed twice more with PBS, and C3 deposition onto beads was analyzed on a BD LSRII flow cytometer and the median fluorescence intensity of the FITC^+^ of all beads was measured.

#### Rapid fluorimetric antibody-mediated cytotoxicity assay (RFADCC)

Antibody-dependent cell-mediated cytotoxicity (ADCC) activity of EBOV GP-reactive IgG or Fab was quantified with an EBOV-adapted modification of the RFADCC assay (Domi et al., 2018, Orlandi et al., 2016). Briefly, a target cell line was made by transfecting 293F cells with a full-length DNA expressing GP from the EBOV-Kikwit isolate followed by transfecting with two separate DNA constructs expressing EGFP and the chimeric CCR5-SNAP tag protein. The new cell line, designated EBOV GPkik-293FS EGFP CCR5-SNAP, expresses EBOV-Kikwit GP on the plasma membrane, EGFP in the cytoplasm and the SNAP-tag CCR5, which can be specifically labeled with SNAP-Surface Alexa Fluor-647 (NEB), on the cell surface (Domi et al., 2018). A human anti-EBOV GP mAb KZ52 (a neutralizing antibody) (IBT) were used as positive control and the unrelated human mAb DENV 2D22 as a negative control. The ADCC activity was quantified by incubating three-fold serial dilutions of mAbs with EBOV GPkik-293FS EGFP CCR5-SNAP target cells for 15 min at ambient temperature and then adding human PBMC as effector cells for 2 hr at 37°C, after which cells were washed once with PBS, fixed with 2% PFA, stained and analyzed with an LSRII Fortessa flow cytometer (BD Biosciences). Data analysis was performed with FlowJo software (Tree Star Inc.). The percentage cytotoxicity of the mAb was determined as the number of target cells losing EGFP (by virtue of ADCC) but retaining the surface expression of CCR5-SNAP.

#### Analysis of viremia by plaque assay

Virus titration was performed in Vero-E6 cells by plaque assay on serum samples collected from ferrets, as previously described (Ilinykh et al., 2016) with some modifications. Briefly, duplicate 10-fold serial dilutions of sera were applied to Vero-E6 cell monolayers in 96 well plates for 1 hr, covered with 100 μL of 0.9% methylcellulose (Sigma) overlay and incubated at 37°C for 6 days. The overlay was removed, cell monolayers were fixed with formalin, washed three times with PBS, and blocked for 1 hr with 5% non-fat dry milk in PBS-T. Plaques were immunostained with rabbit anti-GP primary antibodies (IBT Bioservices) at a 1:5,000 followed by goat-anti rabbit secondary IgG polyclonal HRP-labeled antibody (KPL) at a 1:1,000 dilution in PBS-T. Virus plaques were visualized by staining with a 4CN two component peroxidase substrate system (KPL). The limit of detection was 100 PFU/mL.

#### Serum chemistry markers

Serum samples were tested for concentrations of albumin, amylase, alanine aminotransferase, total bilirubin, alkaline phosphatase, glucose, total protein, blood urea nitrogen, creatinine, phosphorus, calcium (Ca^2+^), sodium (Na^+^), potassium (K+), and globulin by using a VetScan VS2 Chemistry Analyzer with comprehensive Diagnostic Profile Reagent Rotor Package (Abaxis).

#### Single particle electron microscopy

Antibody Fab proteins were obtained by recombinant expression as described above or were generated by digestion of the corresponding IgG with papain (ThermoFisher). Fabs of EBOV-515 or EBOV-520 were added in 5 M excess to EBOV GP ΔTM and allowed to bind overnight at 4°C. Complexes were purified subsequently by size exclusion chromatography on an S200 Increase column (GE HealthCare), then deposited on copper mesh grids coated with carbon and stained 2% uranyl formate. Micrographs were collected using a 120KeV Tecnai Spirit with TVIPS TemCam F416 (4k x 4k) at a defocus of about 1.5e-06 defocus and a dose of 25e-/Å^2^. Micrographs were collected using Leginon (Potter et al., 1999) and processed on Appion (Lander et al., 2009). Particles were picked using DoGpicker (Voss et al., 2009) and aligned with MSA/MRA (Ogura et al., 2003) where excess Fab or blurry particles were removed. An unbinned, clean dataset was deposited into Relion (Scheres, 2012) where 3D classification and refinement was performed. Figures were created in Chimera to compare EBOV complexes and show epitope location.

### Quantification and Statistical Analysis

The descriptive statistics mean ± SEM or mean ± SD were determined for continuous variables as noted. Survival curves were estimated using the Kaplan Meier method and curves compared using the two-sided log rank test with subjects right censored, if they survived until the end of the study. Comparisons of viral titers were performed using a Mann-Whitney U test. ^∗^p < 0.05; ^∗∗^p < 0.01; ^∗∗∗^p < 0.001; ns – non-significant. Statistical analyses were performed using Prism v7.2 (GraphPad).

### Data and Software Availability

The accession numbers for the negative stain EM reconstructions reported in this paper have been deposited to the Electron Microscopy Data Bank under accession numbers EMDB: EMD-7955 and EMD-7956 (see Key Resources Table for details). All relevant data are included with the manuscript; source data for each of the display items is provided in Key Resources Table.

## Supplemental Information

Document S1. Figures S1–S7 and Tables S1–S4

Document S2. Article plus Supplemental Information
