# Supplementary material for: Multifunctional Pan-ebolavirus Antibody Recognizes a Site of Broad Vulnerability on the Ebolavirus Glycoprotein
Source: Immunity. 2018 Aug 21;49(2):363–374.e10. doi: 10.1016/j.immuni.2018.06.018 (PMC6104738; doi:10.1016/j.immuni.2018.06.018)
Supplement: Document S1. Figures S1–S7 and Tables S1–S4 [file mmc1.pdf]

Immunity, Volume *49*

Supplemental Information

Multifunctional Pan-ebolavirus Antibody Recognizes a Site of Broad Vulnerability on the Ebolavirus Glycoprotein

Pavlo Gilchuk, Natalia Kuzmina, Philipp A. Ilinykh, Kai Huang, Bronwyn M. Gunn, Aubrey Bryan, Edgar Davidson, Benjamin J. Doranz, Hannah L. Turner, Marnie L. Fusco, Matthew S. Bramble, Nicole A. Hoff, Elad Binshtein, Nurgun Kose, Andrew I. Flyak, Robin Flinko, Chiara Orlandi, Robert Carnahan, Erica H. Parrish, Alexander M. Sevy, Robin G. Bombardi, Prashant K. Singh, Patrick Mukadi, Jean Jacques Muyembe-Tamfum, Melanie D. Ohi, Erica Ollmann Saphire, George K. Lewis, Galit Alter, Andrew B. Ward, Anne W. Rimoin, Alexander Bukreyev, and James E. Crowe Jr.

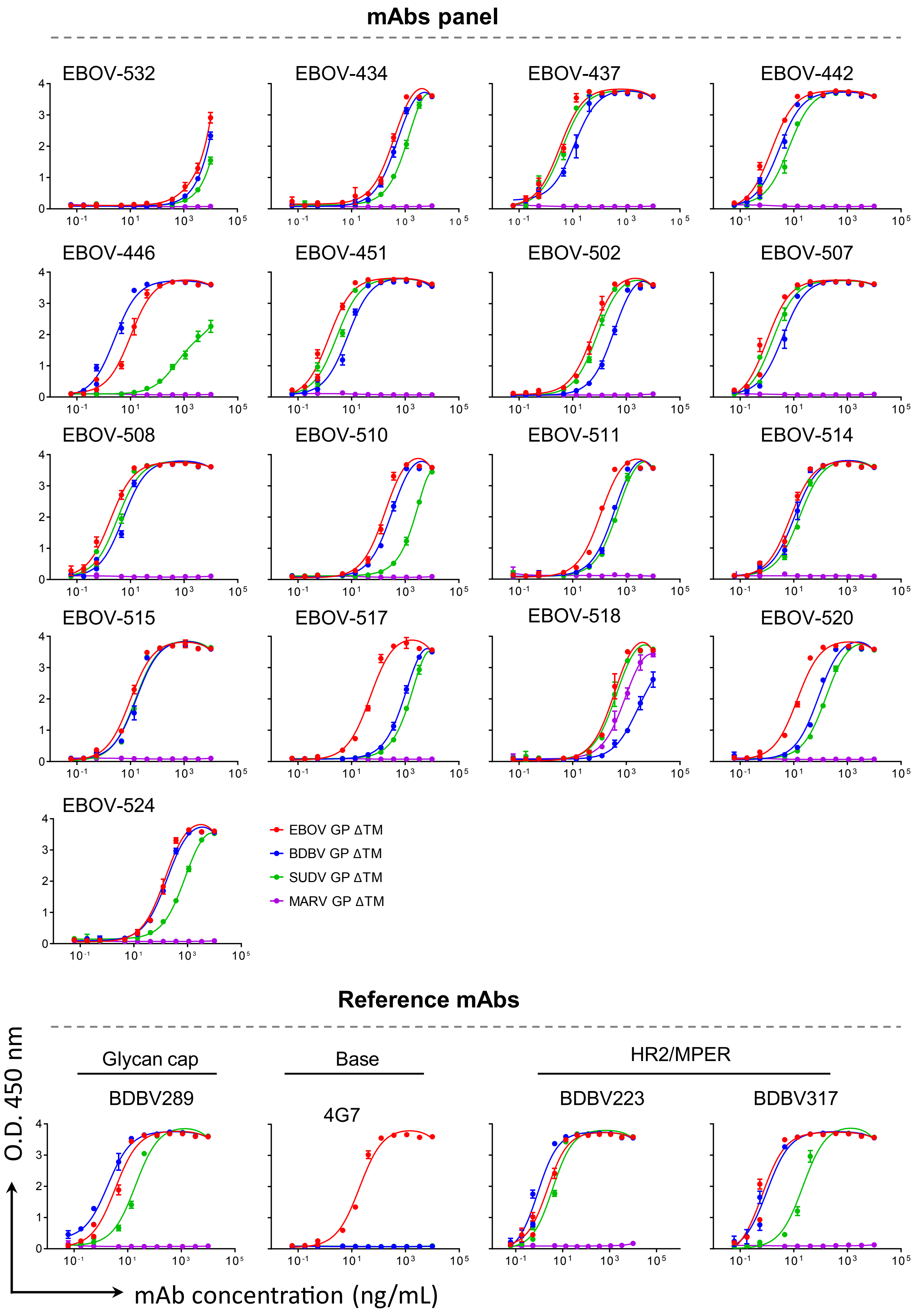
mAbs panel
EBOV-532
EBOV-434
EBOV-437
EBOV-442
EBOV-446
EBOV-451
EBOV-502
EBOV-507
EBOV-508
EBOV-510
EBOV-511
EBOV-514
EBOV-515
EBOV-517
EBOV-518
EBOV-520
EBOV-524
EBOV GP ΔTM
BDBV GP ΔTM
SUDV GP ΔTM
MARV GP ΔTM
Reference mAbs
Glycan cap
Base
HR2/MPER
BDBV289
4G7
BDBV223
BDBV317
O.D. 450 nm
mAb concentration (ng/mL)

Figure S1

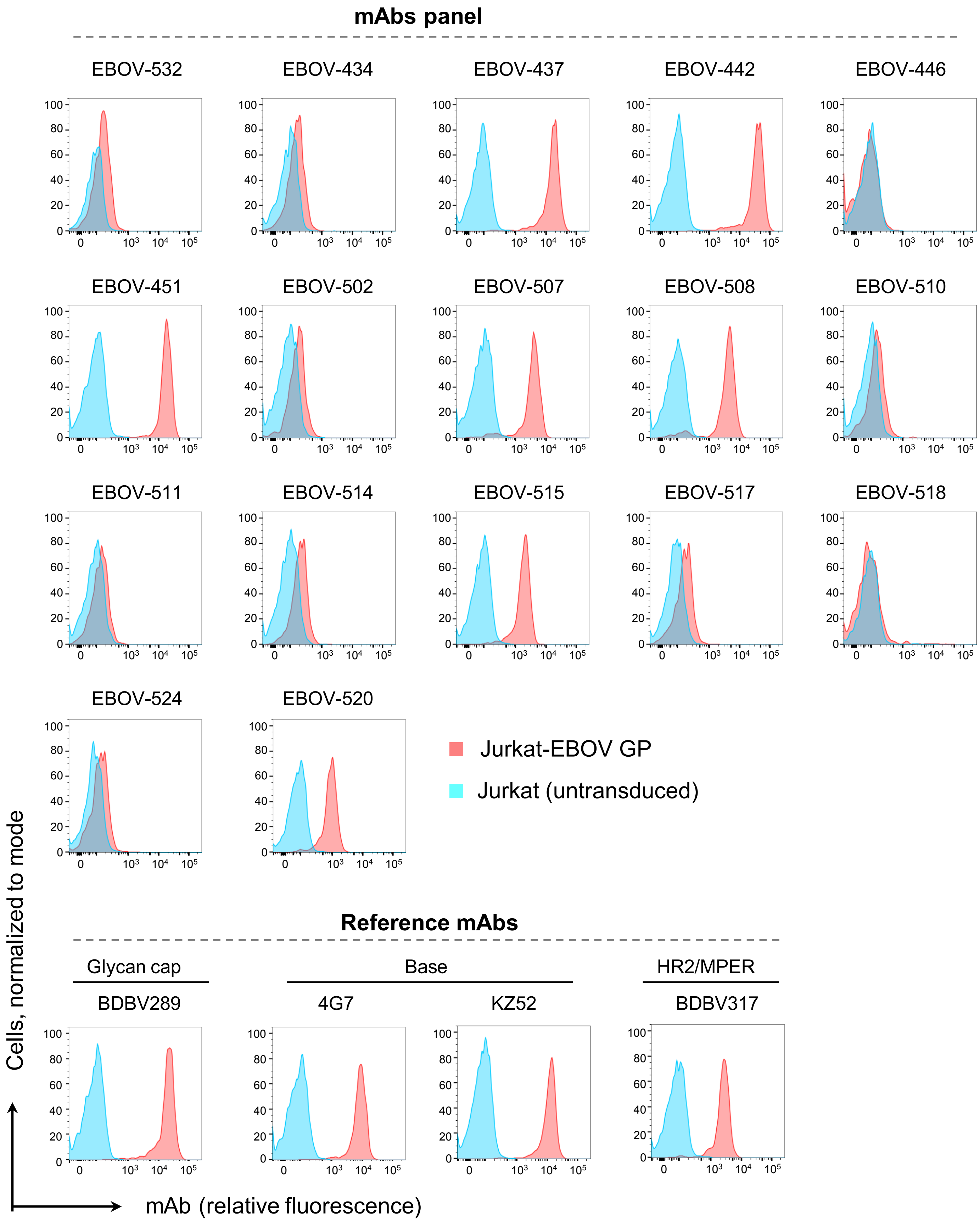
mAbs panel
EBOV-532
EBOV-434
EBOV-437
EBOV-442
EBOV-446
EBOV-451
EBOV-502
EBOV-507
EBOV-508
EBOV-510
EBOV-511
EBOV-514
EBOV-515
EBOV-517
EBOV-518
EBOV-524
EBOV-520
Jurkat-EBOV GP
Jurkat (untransduced)
Reference mAbs
Glycan cap
Base
HR2/MPER
BDBV289
4G7
KZ52
BDBV317
Cells, normalized to mode
mAb (relative fluorescence)

Figure S2

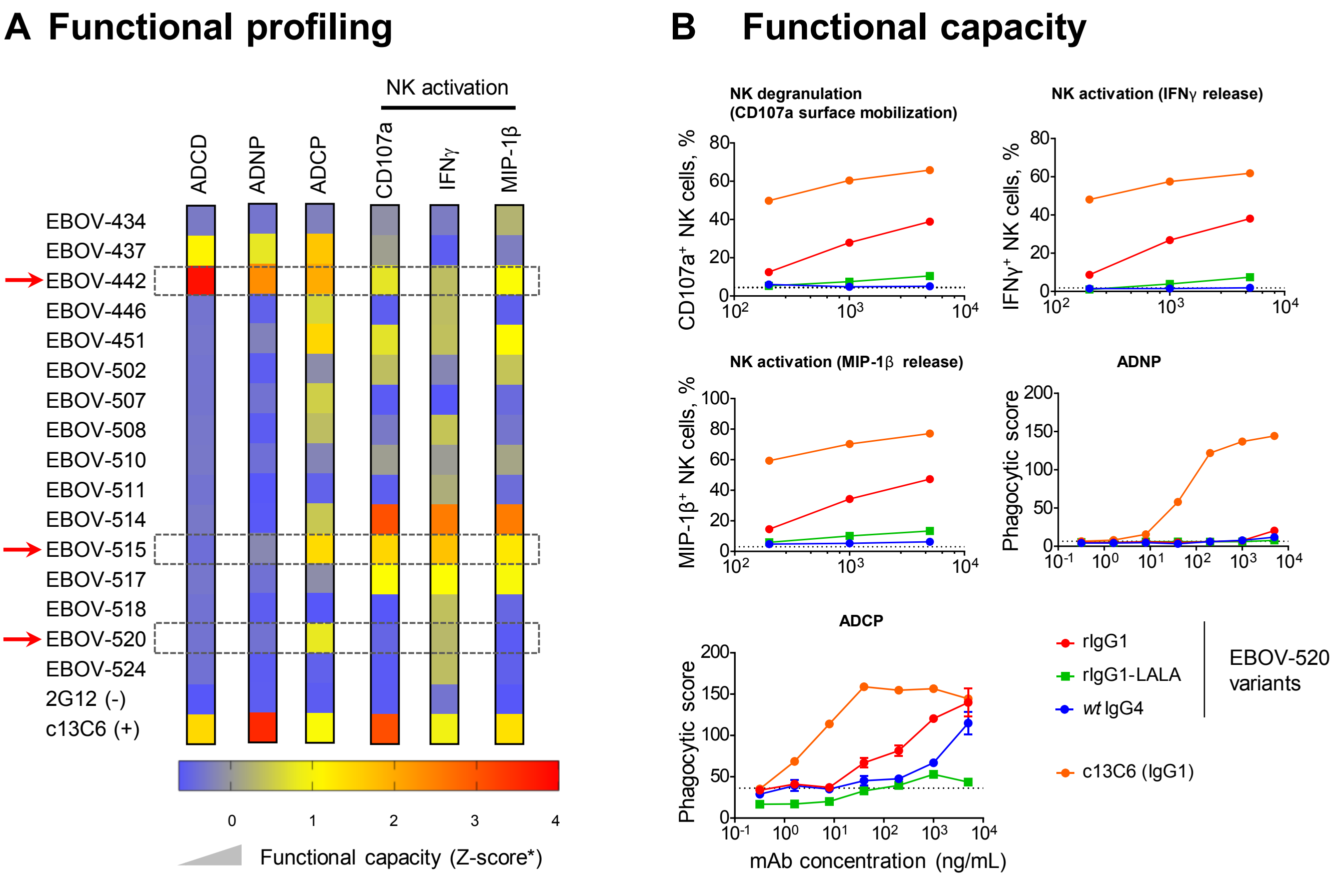
A Functional profiling
NK activation
ADCD
ADNP
ADCP
CD107a
IFNγ
MIP-1β
EBOV-434
EBOV-437
EBOV-442
EBOV-446
EBOV-451
EBOV-502
EBOV-507
EBOV-508
EBOV-510
EBOV-511
EBOV-514
EBOV-515
EBOV-517
EBOV-518
EBOV-520
EBOV-524
2G12 (-)
c13C6 (+)
0
1
2
3
4
Functional capacity (Z-score*)
B Functional capacity
NK degranulation (CD107a surface mobilization)
CD107a⁺ NK cells, %
NK activation (IFNγ release)
IFNγ⁺ NK cells, %
NK activation (MIP-1β release)
MIP-1β⁺ NK cells, %
ADNP
Phagocytic score
ADCP
Phagocytic score
mAb concentration (ng/mL)
rIgG1
rIgG1-LALA
wt IgG4
EBOV-520 variants
c13C6 (IgG1)

Figure S3

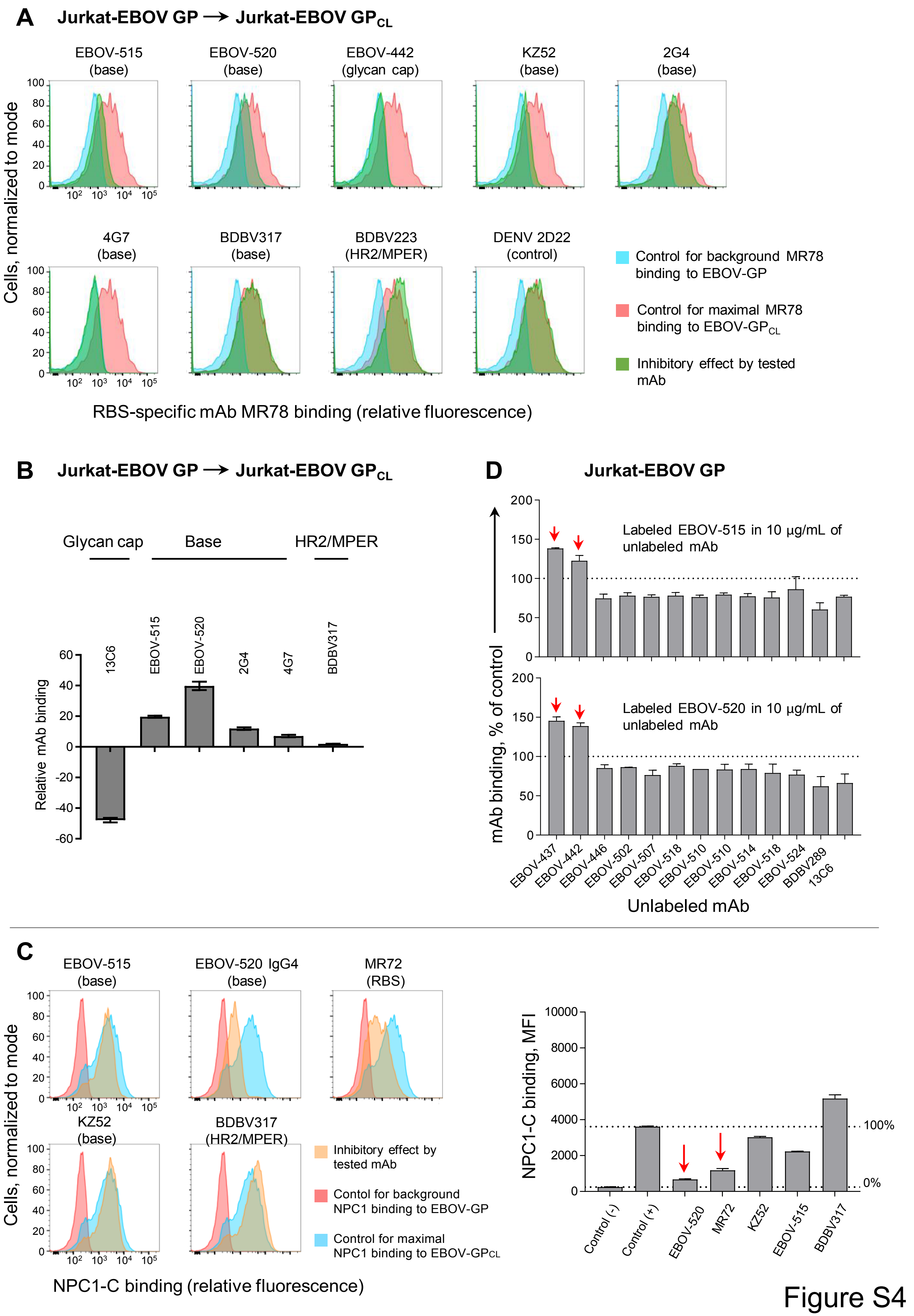
A Jurkat-EBOV GP → Jurkat-EBOV GPCL
EBOV-515 (base)
EBOV-520 (base)
EBOV-442 (glycan cap)
KZ52 (base)
2G4 (base)
4G7 (base)
BDBV317 (base)
BDBV223 (HR2/MPER)
DENV 2D22 (control)
Cells, normalized to mode
Control for background MR78 binding to EBOV-GP
Control for maximal MR78 binding to EBOV-GPCL
Inhibitory effect by tested mAb
RBS-specific mAb MR78 binding (relative fluorescence)
B Jurkat-EBOV GP → Jurkat-EBOV GPCL
Glycan cap
Base
HR2/MPER
13C6
EBOV-515
EBOV-520
2G4
4G7
BDBV317
Relative mAb binding
D Jurkat-EBOV GP
Labeled EBOV-515 in 10 µg/mL of unlabeled mAb
Labeled EBOV-520 in 10 µg/mL of unlabeled mAb
mAb binding, % of control
EBOV-437
EBOV-442
EBOV-446
EBOV-502
EBOV-507
EBOV-518
EBOV-510
EBOV-510
EBOV-514
EBOV-518
EBOV-524
BDBV289
13C6
Unlabeled mAb
C
EBOV-515 (base)
EBOV-520 IgG4 (base)
MR72 (RBS)
KZ52 (base)
BDBV317 (HR2/MPER)
Cells, normalized to mode
Inhibitory effect by tested mAb
Contol for background NPC1 binding to EBOV-GP
Control for maximal NPC1 binding to EBOV-GPCL
NPC1-C binding (relative fluorescence)
NPC1-C binding, MFI
100%
0%
Control (-)
Control (+)
EBOV-520
MR72
KZ52
EBOV-515
BDBV317

Figure S4

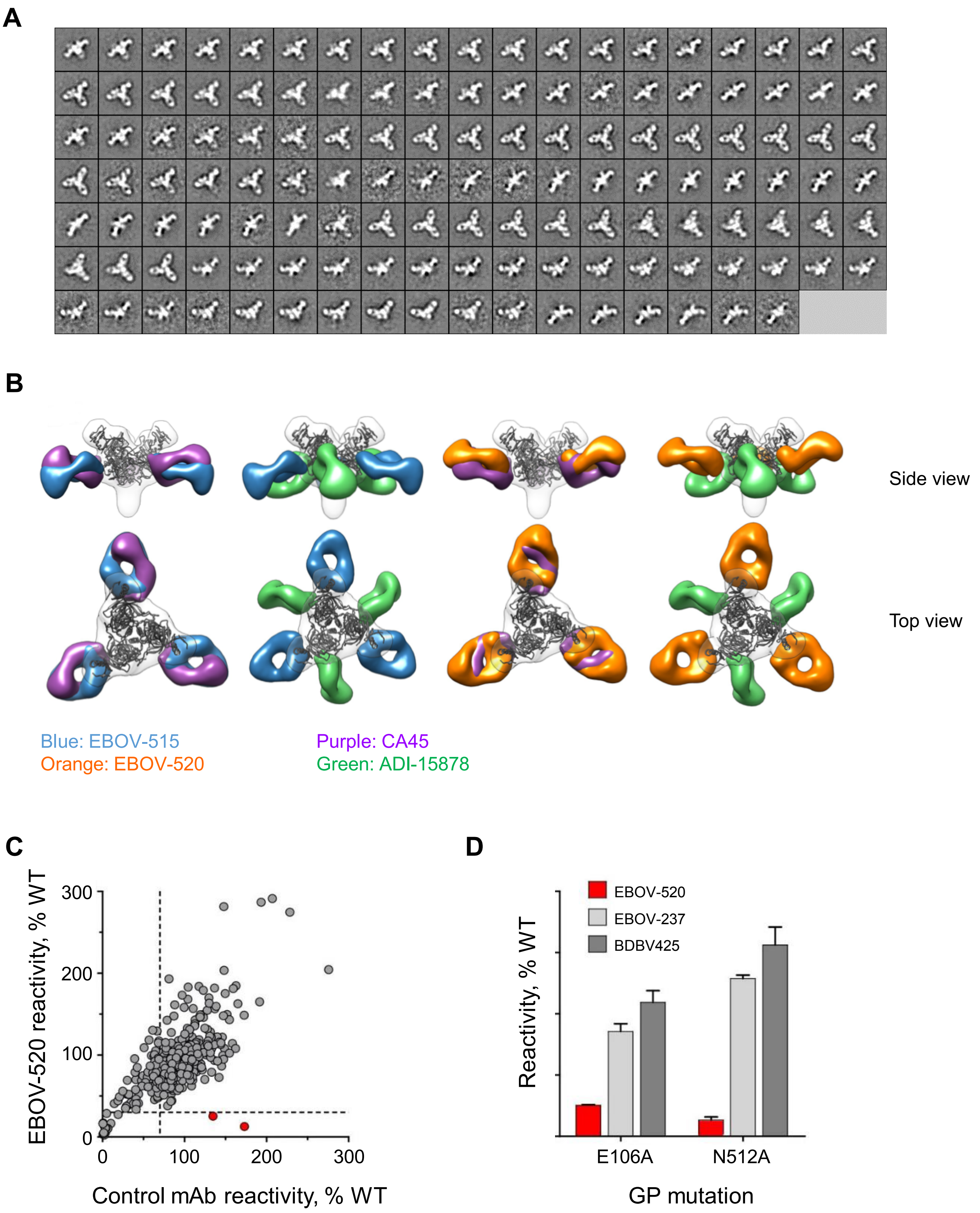
A
B
Side view
Top view
Blue: EBOV-515
Orange: EBOV-520
Purple: CA45
Green: ADI-15878
C
EBOV-520 reactivity, % WT
Control mAb reactivity, % WT
0
100
200
300
D
EBOV-520
EBOV-237
BDBV425
Reactivity, % WT
E106A
N512A
GP mutation

Figure S5

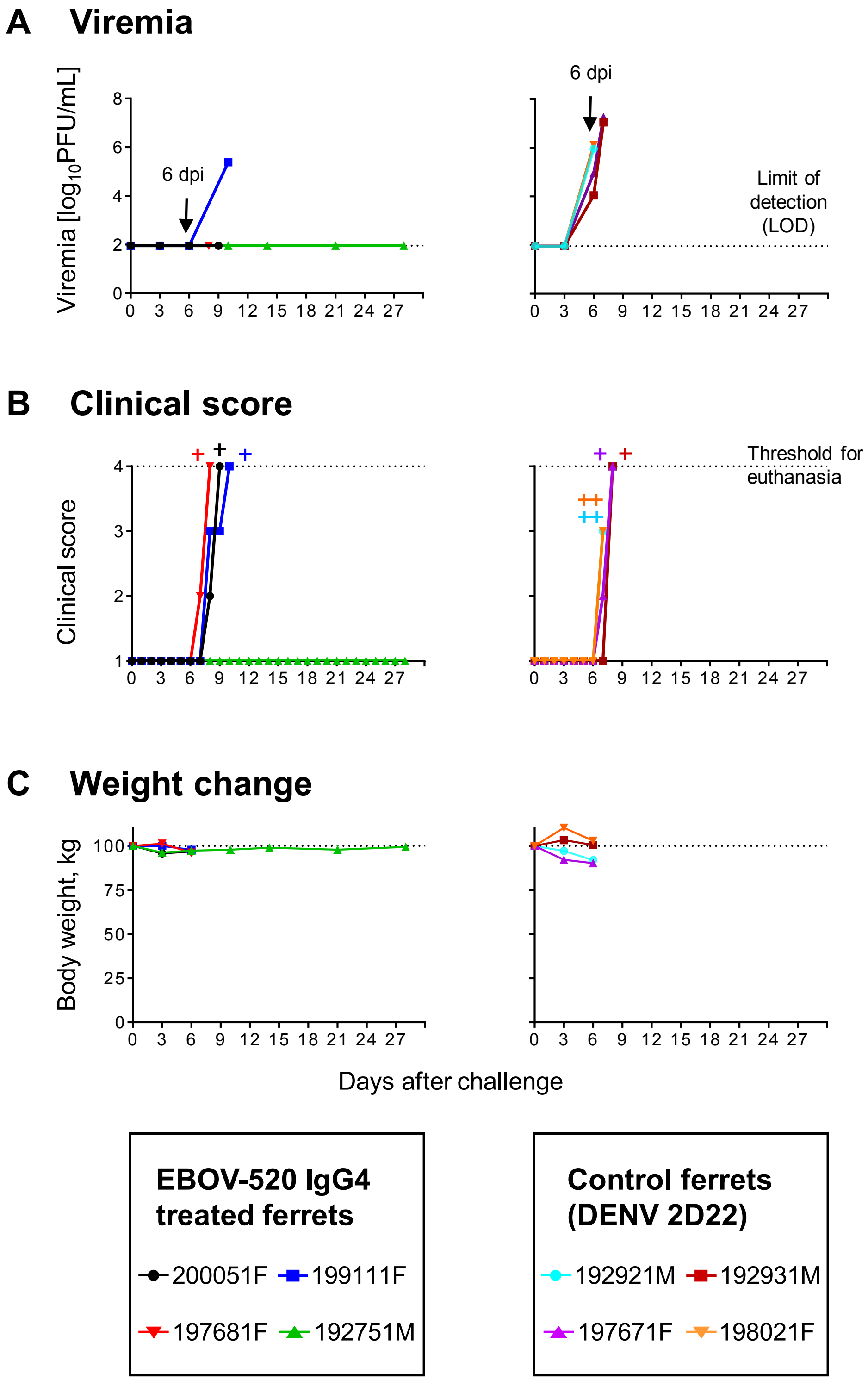
A Viremia
Viremia [log10PFU/mL]
6 dpi
6 dpi
Limit of detection (LOD)
B Clinical score
Clinical score
Threshold for euthanasia
C Weight change
Body weight, kg
Days after challenge
EBOV-520 IgG4 treated ferrets
200051F
199111F
197681F
192751M
Control ferrets (DENV 2D22)
192921M
192931M
197671F
198021F

Figure S6

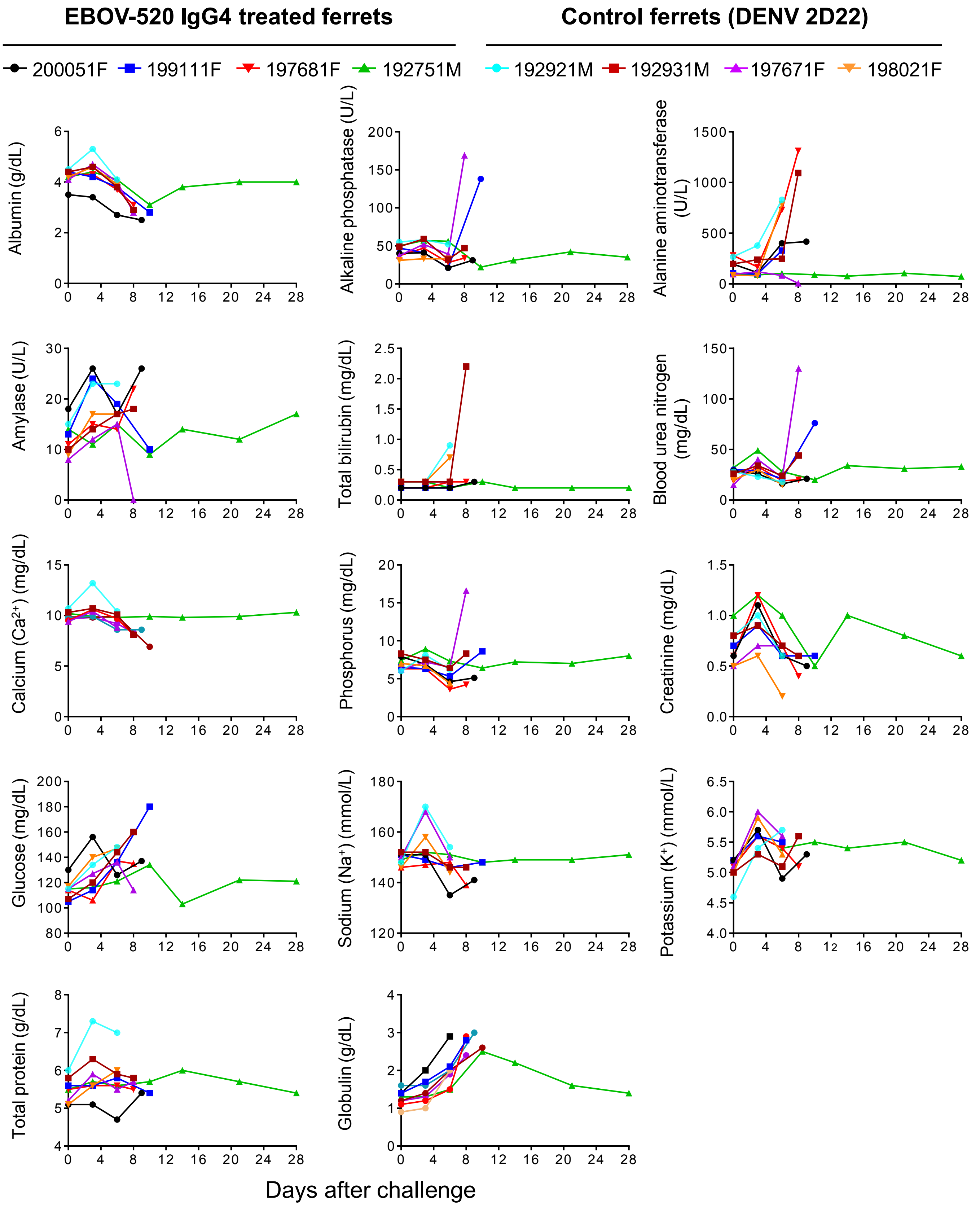

EBOV-520 IgG4 treated ferrets
Control ferrets (DENV 2D22)
200051F
199111F
197681F
192751M
192921M
192931M
197671F
198021F
Albumin (g/dL)
Alkaline phosphatase (U/L)
Alanine aminotransferase (U/L)
Amylase (U/L)
Total bilirubin (mg/dL)
Blood urea nitrogen (mg/dL)
Calcium (Ca^{2+}) (mg/dL)
Phosphorus (mg/dL)
Creatinine (mg/dL)
Glucose (mg/dL)
Sodium (Na^{+}) (mmol/L)
Potassium (K^{+}) (mmol/L)
Total protein (g/dL)
Globulin (g/dL)
Days after challenge

Figure S7

Figure S1. High level of binding of isolated broadly reactive mAbs to EBOV, BDBV, and SUDV GP ΔTM in ELISA. Related to Figure 2.

Binding curves for newly isolated, or previously isolated mAbs to recombinant filovirus GP ΔTM in ELISA. Mean ± SD of four replicates are shown, and data represent one of three independent experiments.

Figure S2. A fraction of broadly reactive mAbs of the panel bind to a cell surface displayed EBOV GP. Related to Figure 2.

Overlay histograms showing mAbs binding to Jurkat- EBOV GP by flow cytometric analysis. Fluorescently labeled mAbs were assessed at 5 μg/mL. Cells were gated for the viable cell population. Data represent one of three independent experiments.

Figure S3. Broadly neutralizing mAbs possess Fc region effector function activity. Related to Figure 3.

(**A**) Heat map of Fc-mediated functional activity for a selected panel of broadly reactive mAbs. Purified mAbs were assessed at 5 μg/mL by the indicated *in vitro* assays, and the results were compared to the controls. The HIV-specific mAb 2G12, and glycan cap-specific mAb c13C6 served as a negative (-) or positive (+) controls, respectively. The red arrow indicates newly identified bNAbs. * Indicates Z-score: $z = (x-\mu)/\sigma$, where x is raw score (a phagocytic score, or MFI, or percent activated cells that determined as described in the Methods Details section), μ is the mean of the population, and σ is the standard deviation of the population.

(**B**) Functional capacity curves for IgG heavy chain engineered variants of bNAb EBOV-520. Mean ± SD of duplicates are shown for ADCP and ADNP assays. NK activation data are representative for one of two biological replicates.

Figure S4. EBOV-515 and -520 use several mechanisms to facilitate virus neutralization. Related to Figure 4 and Figure 5.

(**A**) Capacity of mAbs to inhibit RBS exposure after GP to GP_{CL} cleavage using Jurkat-EBOV GP. Cells were pre-incubated with 40 μg/mL of indicated mAb before cleavage with thermolysin. RBS exposure was determined by binding of fluorescently labeled RBS-specific mAb MR78. Binding

of MR78 mAb alone to EBOV GP (blue) or to EBOV GP_{CL} (red) served as negative or positive controls, respectively.

(**B**) Fold change in mAb binding after EBOV GP to EBOV GP_{CL} cleavage using cell surface display. Each mAb was tested at 5 µg/mL. Positive values indicate increase in binding, and negative values indicate decrease, respectively.

(**C**) Capacity to inhibit receptor binding. Binding of soluble NPC1-C to GP_{CL} was assessed after incubation of Jurkat-EBOV GP_{CL} with individual mAbs. EBOV-520 and MR72, but not EBOV-515, KZ52, or BDBV317 mAbs inhibit NPC1-C binding. Binding of NPC1-C to Jurkat-EBOV GP_{CL} or Jurkat-EBOV cells served as controls. MAbs were assessed at 50 µg/mL.

(**D**) Identification of mAbs that cooperated in binding with EBOV-515 or -520 mAbs. A mixture of individual unlabeled non-competing mAb and fluorescently-labeled EBOV-515 or -520 mAb was incubated with Jurkat-EBOV GP, followed by flow cytometric analysis. Binding of fluorescently-labeled EBOV-515 or-520 alone served as a control to define 100% binding activity (dotted line). Binding to untransduced Jurkat cells served as a negative control for the assay background. Red arrows indicate two mAbs that cooperated in binding with EBOV-515 or -520. Mean ± SD of triplicates are shown. Data shown are representative of 2-3 experiments.

Figure S5. EBOV-515 and -520 recognize distinct vulnerable epitopes in the ebolavirus GP base region. Related to Figure 6.

(**A**) 2D class average of EBOV-520 Fab bound to EBOV GP ΔTM determined by single particle EM.

(**B**) 3D reconstructions of Fab/EBOV GP ΔTM complexes from single particle EM studies. Fab of EBOV-515 (blue), EBOV-520 (orange), CA45 (violet), or ADI-15878 (green) were superimposed to compare the angle of approach for newly identified (EBOV-515 and -520) and previously reported (CA45 and ADI-15878) broadly neutralizing base mAbs. A model of EBOV GP ΔTM trimer was fitted into the density.

(**C-D**) Shotgun mutagenesis epitope mapping of EBOV-520. (**C**) Identified two critical clones E106A and N512A (shown in red) that showed specifically reduced binding for EBOV-520 Fab (<30% of binding to *wt* EBOV GP), but a high level of binding to the control mAb. (**D**) Mutation to of either E106 and N512 to alanine reduced EBOV-520 binding (red bars) but did not affect

binding of control mAbs EBOV237 or BDBV425 (gray bars). Error bars represent the mean and range (half of the maximum minus minimum values) of at least two replicates.

Figure S6. EBOV-520 mediates protection against BDBV in ferret challenge model. Related to Figure 7.

Groups of male and female ferrets (denoted with M or F suffix to animal number) were inoculated with 1,000 PFU of BDBV, treated on 3 and 6 dpi with 18 mg of the EBOV-520 IgG4 or control DENV 2D22 mAb by i.p. injection, and monitored for 28 days.

(**A**) Viral burden measured in blood using plaque assay is shown. Mean of technical duplicates is shown.

(**B**) Clinical score is shown. +, diseased animal that was euthanized as mandated by IACUC. ++, animal found dead (8 dpi) between observations prior to reaching the pre-determined clinical score and despite the increased observation schedule.

(**C**) Body weight change is shown.

Data represent one experiment.

Figure S7. Analysis of blood chemistry markers in BDBV-challenged ferrets. Related to Figure 7.

Blood chemistry changes in ferrets treated with EBOV-520 IgG4 or DENV 2D22 as a control. Data represent one experiment.

Table S1. Germline origin genes and variable region analysis of newly identified broadly reactive human mAbs. Related to Figure 1

mAb*	Heavy chain variable gene sequence						Light chain variable sequence				
	V-gene and allele	V-region nucleotide homology to V-gene, %	D-gene and allele	J-gene and allele	CDR3 amino acids (aa)	CDR3 length (aa)	V-gene and allele	V-region nucleotide homology to V gene, %	J-gene and allele	CDR3 amino acids (aa)	CDR3 length (aa)
EBOV-434	1-2*02	99	3-10*01	4*03	ARDSGELLFVGSDV	14	1-12*01 or 1-12*02 or 1D-12*02	98	2*01	QQANSFPQT	9
EBOV-437	1-69*06	95	3-10*01	J6*03	ARGPPLRGERSWFGESEKYDYFYMDV	26	N/A	N/A	N/A	N/A	N/A
EBOV-442	3-15*01	91	3-16*01	6*03	ATGSGKGPSASFGESYYYYDFINV	24	3-20*01	96	1*01	HQYESSPWT	9
EBOV-446	1-2*02 or 1-2*04	95	4-17*01	4*02	ARGRRHGAYVD	11	1-9*01	96	5*01	QQLNFYLGGLT	11
EBOV-451	3-20*01	92	2-21*01	4*02	VSWGERYDAYFDY	13	3-20*01	96	2*01	QQYGSSPYT	9
EBOV-502	3-23*04	93	5-18*01	6*02	AKDAQQETDIVYFYYYDGMDV	21	N/A	N/A	N/A	N/A	N/A
EBOV-507	1-2*02	90	2-8*02	3*01	WIWFRSETFDF	11	1-17*03	96	1*01	LQHNTYLT	8
EBOV-508	1-46*01 or 1-46*02 or 1-46*03	95	3-16*02	4*02	VSFQFYFDY	9	1-17*03	99	1*01	LQHNSYPWT	9
EBOV-510	4-30-4*01	93	4-17*01	6*02	ARESDGDPSRLYFYFAMDV	19	2-14*01	96	2*01 or 3*01 or 3*02	SSYTSNTTLV	10
EBOV-511	4-39*01	92	3-3*01	4*02	ARHLAPISGVIFIPSFFDS	19	3-15*01	97	4*01	QQYNDWPPRLT	11
EBOV-514	4-30-4*01	91	2-2*01	6*03	ARDKAQAYGLLYHYHTDV	18	3-15*01	96	2*01	QYYNDWPPGYT	11
EBOV-515	4-31*03 or 4-31*07	93	3-22*01	4*02	ARESSWVSELGRDN	14	3-15*01	97	1*01	QQYNNWPRT	9
EBOV-517	3-7*01	96	2-2*01	6*03	ARGASIEVEILYYYHMDV	18	3-15*01	94	4*01	QQYHTWPPLT	10
EBOV-518	2-5*09	92	3-16*02	4*02	AHSGGLVAGAFDY	13	4-1*01	94	1*01	QQYYNSPRT	9
EBOV-520	4-59*01	93	5-12*01	6*02	ARGAWNVATVYYYYGMDV	18	3-20*01	97	2*01	QQYGNSLYT	9
EBOV-524	3-30*02 or 3-30-5*02	90	2-2*01	6*03	AKDVLDCSRADCFIYYYYMDV	21	1-12*01 or 1-12*02 or 1D-12*02	83	4*01	QQGNRIPLS	9

N/A – not available.

* - MAbs EBOV-434 to EBOV-451 were isolated from a survivor of the West African 2013-2016 EVD epidemic; MAbs EBOV-502 to EBOV-24 were isolated from a survivor of the 2014 EVD outbreak in the DRC.

Table S2. Binding capacity of newly isolated broadly-reactive mAbs, or previously described mAbs, assessed by ELISA. Related to Figure 2

**mAb origin**	**mAb ID**	**Isotype**	**Binding, EC_{50} ng/mL** (95% CI)			
			**EBOV GP ΔTM**	**BDBV GP ΔTM**	**SUDV GP ΔTM**	**MARV GP ΔTM**
Survivor of the West African 2013-2016 EVD epidemic	EBOV-434	IgG1	**304** (237 to 389)	**415** (354 to 486)	**1,114** (977 to 1,268)	>
	EBOV-437	IgG1	**3** (2 to 4)	**11** (8 to 14)	**4** (3 to 5)	>
	EBOV-442	IgG1	**1** (1 to 2)	**3** (2 to 3)	**6** (5 to 8)	>
	EBOV-446	IgG1	**9** (8 to 12)	**2** (2 to 3)	**722** (584 to 892)	>
	EBOV-451	IgG1	**1** (1 to 2)	**7** (5 to 9)	**3** (2 to 3)	>
Survivor of the 2014 EVD outbreak in the DRC	EBOV-502	IgG1	**50** (41 to 61)	**285** (241 to 338)	**73** (62 to 85)	>
	EBOV-507	IgG1	**1** (1 to 1)	**4** (3 to 5)	**2** (1 to 2)	>
	EBOV-508	IgG1	**2** (1 to 2)	**5** (43 to 6)	**3** (2 to 4)	>
	EBOV-510	IgG1	**143** (112 to 182)	**261** (216 to 314)	**3,091** (2,783 to 3,433)	>
	EBOV-511	IgG4	**94** (74 to 119)	**289** (232 to 359)	**387** (323 to 463)	>
	EBOV-514	IgG1	**7** (5 to 8)	**10** (8 to 12)	**15** (12 to 18)	>
	EBOV-515	IgG1	**9** (7 to 11)	**15** (11 to 19)	**14** (11 to 18)	>
	EBOV-517	IgG1	**42** (33 to 53)	**865** (760 to 984)	**1,694** (1,506 to 1,906)	>
	EBOV-518	IgG3	**283** (220 to 365)	**2,626** (2,138 to 3,225)	**352** (282 to 440)	**780** (668 to 958)
	EBOV-520	IgG4	**12** (10 to 15)	**72** (58 to 88)	**136** (118 to 156)	>
	EBOV-524	IgG4	**123** (100 to 151)	**146** (125 to 170)	**701** (636 to 774)	>
Reference mAb	BDBV-289	IgG1	**3** (2 to 4)	**2** (1 to 2)	**18** (14 to 22)	>
	BDBV-317	IgG1	**<1**	**<1**	**19** (15 to 25)	>
	BDBV-223	IgG3	**2** (2 to 3)	**<1**	**3** (3 to 5)	>
	4G7	IgG1	**18** (14 to 22)	>	>	>
	KZ52	IgG1	**14** (11 to 18)	>	>	>

">" Indicates binding was not detected, even at the highest concentration tested of 10,000 ng/mL

Table S3. Neutralizing capacity of newly isolated broadly-reactive mAbs, or previously described mAbs. Related to Figure 2

**mAb origin**	**mAb ID**	**Neutralization, IC_{50} ng/mL** (95% CI)		
		**EBOV**	**BDBV**	**SUDV**
Survivor of the West African 2013-2016 EVD epidemic	EBOV-434	>	>	>
	EBOV-437	**8,660***	>	**27,030***
	EBOV-442	**467** (321 to 679)	**1,489** (861 to 2,577)	**38,330***
	EBOV-446	>	>	>
	EBOV-451	>	>	>
Survivor of the 2014 EVD outbreak in the DRC	EBOV-502	>	>	>
	EBOV-507	>	>	>
	EBOV-508	>	>	>
	EBOV-510	>	>	>
	EBOV-511	>	>	>
	EBOV-514	>	>	>
	EBOV-515	**1,224** (769 to 1,950)	**1,458** (1,070 to 1987)	**891** (653 to 1,217)
	EBOV-517	>	>	>
	EBOV-518	>	>	>
	EBOV-520	**5,738** (3,818 to 8,624)	**3,810** (2,701 to 5,375)	**6,318** (3,636 to 10,980)
	EBOV-524	>	>	>
Reference mAb	BDBV-289**	**588**	**32**	>
	BDBV-317**	**4,400**	**100**	>
	BDBV-223**	**100**	**20**	>
	4G7**	**135**	>	>
	KZ52**	**400**	>	>

* Incomplete (<100%) virus neutralization at the highest mAb concentration tested (200 µg/mL).
">" Neutralization was not detected at the highest mAb concentration tested (200 µg/mL).
** Neutralization data from previous reports that are included here for comparative purposes.

Table S4. Fc-mediated functional capacity of isolated broadly-reactive mAbs*. Related to Figure 3

mAb	Functional assay, z-score*					
	ADCD	ADNP	ADCP	NK CD107	NK IFN-γ	NK MIP-1β
EBOV-434	-0.29	-0.23	-0.60	-0.27	-1.02	0.08
EBOV-437	1.10	0.78	1.66	-0.09	-1.51	-0.55
EBOV-442	3.80	2.33	1.97	0.71	-0.03	0.95
EBOV-446	-0.36	-0.40	0.51	-0.82	-0.03	-0.91
EBOV-451	-0.34	-0.12	1.47	0.68	0.03	1.04
EBOV-502	-0.36	-0.44	-0.44	0.26	-0.87	0.28
EBOV-507	-0.35	-0.25	0.39	-0.86	-1.59	-0.78
EBOV-508	-0.33	-0.45	0.17	-0.51	0.09	-0.66
EBOV-510	-0.31	-0.28	-0.54	-0.10	-0.54	-0.09
EBOV-511	-0.37	-0.49	-0.98	-0.82	-0.27	-0.76
EBOV-514	-0.32	-0.48	0.30	3.05	2.53	2.53
EBOV-515	-0.41	-0.06	1.42	1.48	1.71	1.26
EBOV-517	-0.34	-0.27	-0.43	0.97	0.97	0.93
EBOV-518	-0.34	-0.44	-1.10	-0.89	0.03	-0.83
EBOV-520	-0.37	-0.26	0.72	-0.74	-0.11	-0.97
EBOV-524	-0.38	-0.45	-0.99	-0.87	-0.03	-0.90
Neg. control	-0.63	-0.44	-1.04	-0.86	-1.16	-1.01
Pos. control	1.44	3.52	0.93	3.08	0.80	1.34

* Z-score (z) = (x-μ)/σ, where x is raw score (a phagocytic score, or MFI, or percent activated cells that determined as described in the Methods Details section), μ is the mean of the population, and σ is the standard deviation of the population.
